# Comparison of ready-to-eat “organic” antimicrobials, sodium bisulfate, and sodium lactate, on *Listeria monocytogenes* and the indigenous microbiome of organic uncured beef frankfurters stored under refrigeration for three weeks

**DOI:** 10.1371/journal.pone.0262167

**Published:** 2022-01-20

**Authors:** Aaron R. Bodie, Dana K. Dittoe, Kristina M. Feye, Carl J. Knueven, Christina Ovall, Steven C. Ricke

**Affiliations:** 1 Meat Science and Animal Biologics Discovery, Animal and Dairy Sciences, University of Wisconsin, Madison, Wisconsin, United States of America; 2 Cell and Molecular Biology, University of Arkansas, Fayetteville, Arkansas, United States of America; 3 Jones-Hamilton Co., Walbridge, Ohio, United States of America; University of Sassari, ITALY

## Abstract

*Listeria monocytogenes* has been implicated in several ready-to-eat (RTE) foodborne outbreaks, due in part to its ability to survive under refrigerated conditions. Thus, the objective of this study was to evaluate the effects of sodium bisulfate (SBS), sodium lactate (SL), and their combination as short-duration antimicrobial dips (10-s) on *L*. *monocytogenes* and the microbiome of inoculated organic frankfurters (8 Log_10_ CFU/g). Frankfurters were treated with tap water (TW), SBS0.39%, SBS0.78%, SL0.78%, SL1.56%, SBS+SL0.39%, SBS+SL0.78%. In addition, frankfurters were treated with frankfurter solution water (HDW)+SBS0.78%, HDW+SL1.56%, and HDW+SBS+SL0.78%. After treatment, frankfurters were vacuum packaged and stored at 4°C. Bacterial enumeration and 16S rDNA sequencing occurred on d 0, 7, 14, 21. Counts were Log_10_ transformed and calculated as growth potential from d 0 to d 7, 14, and 21. Data were analyzed in R using mixed-effects model and One-Way ANOVA (by day) with differences separated using Tukey’s HSD at P ≤ 0.05. The 16S rDNA was sequenced on an Illumina MiSeq and analyzed in Qiime2-2018.8 with significance at P ≤ 0.05 and Q ≤ 0.05 for main and pairwise effects. An interaction of treatment and time was observed among the microbiological plate data with all experimental treatments reducing the growth potential of *Listeria* across time (P < 0.0001). Efficacy of treatments was inconsistent across time; however, on d 21, SBS0.39% treated franks had the lowest growth potential compared to the control. Among diversity metrics, time had no effect on the microbiota (P > 0.05), but treatment did (P < 0.05). Thus, the treatments potentially promoted a stable microbiota across time. Using ANCOM, *Listeria* was the only significantly different taxa at the genus level (P < 0.05, W = 52). Therefore, the results suggest incorporating SBS over SL as an alternative antimicrobial for the control of *L*. *monocytogenes* in organic frankfurters without negatively impacting the microbiota. However, further research using multiple *L*. *monocytogenes* strains will need to be utilized in order to determine the scope of SBS use in the production of RTE meat.

## Introduction

At time of purchase, fully cooked, ready-to-eat (RTE) meats are assumed safe for consumption without additional preparation, and have a recommended shelf life of two weeks [1]. However, processed RTE products such as deli meats, frankfurters, canned meats (tuna, chicken, and spam), and jerky can be contaminated with *Listeria monocytogenes*, a foodborne pathogen capable of withstanding various stress environments [2–4]. A significant reason why *L*. *monocytogenes* is prominent in these products is its ability to survive a broad range of temperatures, 40 °C to -20 °C, including refrigeration and freezing which may increase the chance of consumers ingesting viable cells [5, 6]. As such, a significant proportion of foodborne disease outbreaks in the U.S. have been associated with RTE products [7, 8]. In the past 3 years, *L*. *monocytogenes* outbreaks associated with RTE meat products have occurred more frequently, with 4 of the 7 more recent *L*. *monocytogenes* outbreaks since 2018 being attributed directly to the consumption of RTE products [8].

The U.S. Food and Drug (FDA) administration’s risk assessment models have estimated that RTE deli meats and non-reheated franks have the highest risk of listeriosis per serving [9]. The risk is directly attributed to *L*. *monocytogenes* contamination that occurs during post-production processes such as peeling, sorting, loading, packaging, and slicing of RTE products at the processor and consumer level [9, 10]. With the increase in frequency of *L*. *monocytogenes* outbreaks and potential for contamination during post-production processes, it is evident that additional efforts must be taken to ensure the safety of RTE meats such as frankfurters.

Previously published work by Bodie et al. [11] determined that the inorganic acid sodium bisulfate (SBS) and nisin applied as short antimicrobial dips were effective strategies at reducing *L*. *monocytogenes* EDG-e on organic uncured beef franks. In continuation with that effort to identify unique multi-hurdle interventions for mitigating *L*. *monocytogenes* (EDG-e) on organic uncured beef franks, two acidic antimicrobials, SBS and sodium lactate (SL), were identified for use as 10-s short duration antimicrobial dips. The inorganic acid, SBS, has been Generally Recognized as Safe (GRAS) according to the FDA, regarded as “natural” by the Grocery Manufacturers Association, termed a safer alternative choice as an antimicrobial by the Environmental Protection Agency, is considered safe and suitable ingredient in the production of meat, poultry, and eggs according to the United States Department of Agriculture (USDA), and is effective against multiple pathogens across various matrices [11–18]. In addition, SL, a synthetic (non-natural) antimicrobial food additive, has been used for the past three decades as an RTE ingredient effectively controlling *L*. *monocytogenes* contamination and serving as a flavor enhancer in processed meat products [19–22]. In 2016, SL was added to the list of approved synthetic compounds to use in “organic” labeled products [23].

**Fig 1:**
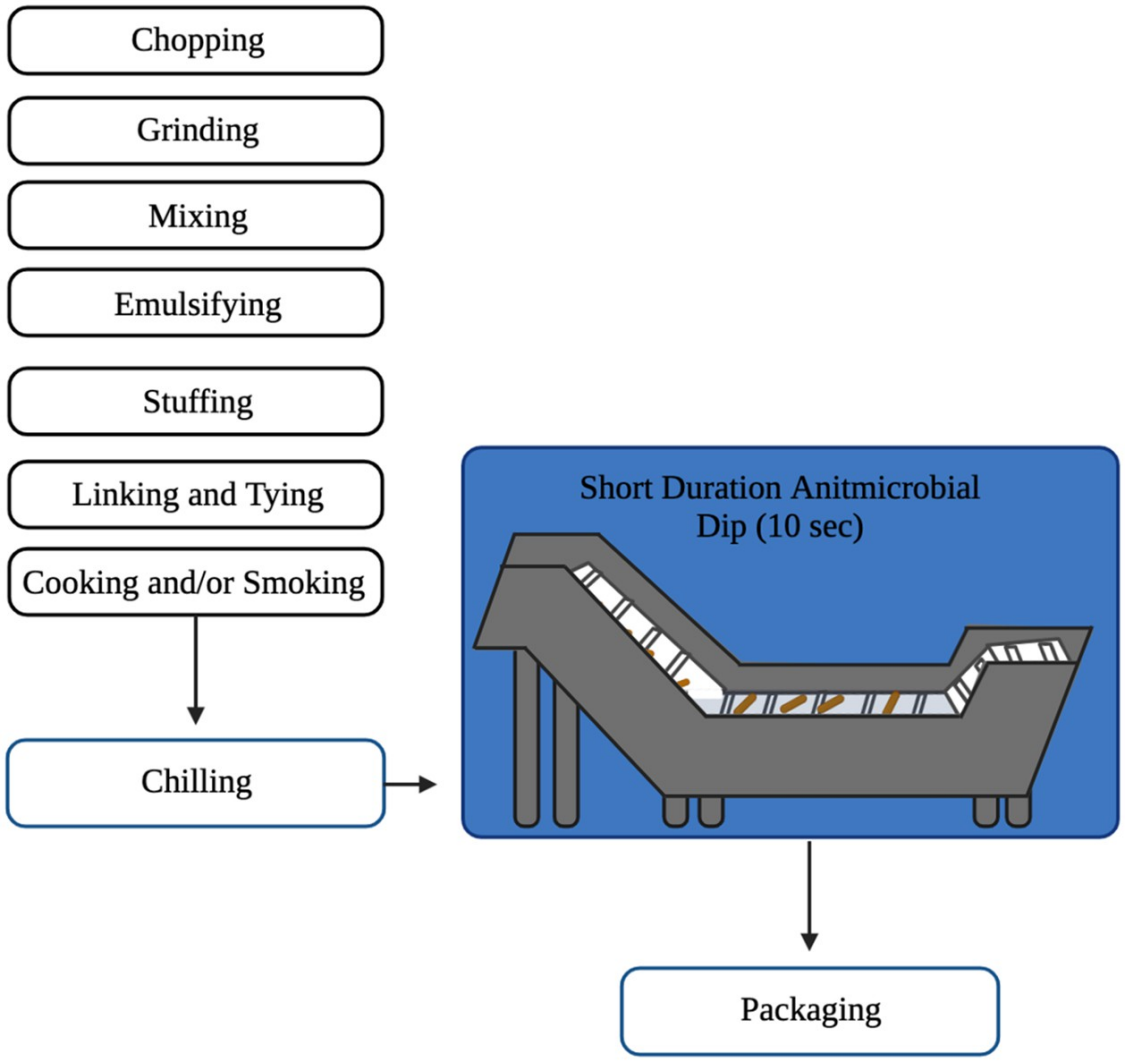
Proposed application of short duration antimicrobial dips during the production of frankfurters. Antimicrobial dips would be applied as 10-s dip after cooling prior to packaging. Therefore, these applied antimicrobials would not be directly introduced into the batter and not considered as an ingredient. Created with BioRender.com.

With the increased demand and consumption of organic and “natural” food items in the U.S., chemical and synthetic preservatives commonly used in the formulation of nonorganic meat products may not be applicable for organic RTE meat products [1, 24]. Therefore, alternative options that can control the growth of *L*. *monocytogenes* without adversely affecting the sensory characteristics and shelf life of RTE meat are becoming more critical. One potential alternative is to apply a multi-hurdle approach employing an antimicrobial short duration dip prior to packaging (after cooking and cooling) to mitigate the contamination of RTE meats such as franks and possibly extend shelf-life (Fig 1). Thus, these antimicrobials would not be directly incorporated into RTE frankfurter batter prior to lethality steps (cooking) but utilized after cooling or cellulose casings are removed just prior to packaging. This application of an antimicrobial dip prior to packaging could allow integrators an additional route to mitigate *L*. *monocytogenes* contamination and the opportunity to utilize a broader range of antimicrobials during organic RTE meat production.

The overall objective of the current study was to determine the effect of two antimicrobials such as SBS, SL, and their combination as short duration antimicrobial dips (10 s) on reducing *L*. *monocytogenes* EDG-e on *L*. *monocytogenes* inoculated organic uncured beef franks during a 21-d shelf-life study. In parallel to mitigating *L*. *monocytogenes*, the current study aimed at investigating the effect of these antimicrobials on the microbiome (16S rDNA) of the inoculated organic uncured beef franks. As there is limited knowledge on the microbiota of these organic uncured beef frankfurters and how they respond to short duration dips using these antimicrobials, this research, to the best of our knowledge, is the first insight into that response in the U.S.

## Materials and methods

### Determination of antimicrobial concentrations

Prior to the onset of the current study, treatment concentrations were determined via Minimum Inhibitory and Minimum Bactericidal Concentration (MIC and MBC, respectively; S1 Fig) based on the methodology described by Bodie et al. [11]. Briefly, an overnight culture of *L*. *monocytogenes* EDG-e was grown aerobically on Oxford agar (Oxoid, Nepean, ON, Canada) at 37 °C for 24 hours. Subsequently, a 100 μL of Tryptic Soy Broth (TSB; Becton, Dickinson and Company, Sparks, Maryland, USA) was transferred into a 96 well microplate. A single colony was selected and aseptically transferred into each well of 100 μL of TSB followed by aerobic incubation at 37 °C for 18 hours to allow for growth.

The MIC was determined for both SBS and SL in the current study. The antimicrobials were arranged in 20 mL of TSB, at 25% and SL at 25% weight to volume. Subsequently, 1:2 dilutions were attained by adding 100 μL of TSB to subsequent wells in a 96-well microplate from 25% to 0.19% of SBS, SL, and their combination. *Listeria* was pin replicated into treatment plates using a 96 pin replicator. Immediately following pin replication, the microplates were incubated for 18 hours at 37°C under aerobic conditions. After incubation, 10 μL of 0.1% resazurin sodium salt (Sigma-Aldrich; St. Louis, MO, USA) was added to each well and allowed to incubate at 37 °C for an additional 3 h. The microplates were observed visually for bacterial growth with viable growth being indicated as purple and no growth being indicated as pink [25].

The MBC was determined by spread plating the entirety of the well (100 μL) determined to be the MIC and the wells containing the contents of concentrations directly above and below the MIC onto Oxford Agar. The concentrations were plated in triplicate and incubated under the previously described conditions to determine the MBC. The concentration plated with no growth after 24 hours of incubation were determined to be the MBC. The procedure was repeated three times in its entirety to determine MIC and MBC.

### Frankfurter procurement and *Listeria* screening

“Organic, all-natural” beef frankfurters (uncured, no-nitrate or nitrite-added, no preservatives, no by-products, fully cooked, vacuum packaged; S1 Table) with an average weight of 47 g were procured from a commercial retailer in northwest Arkansas and transported on ice to the Center for Food Safety at the University of Arkansas (Fayetteville, AR, USA) on three separate occasions (3 individual trials). There was a sample size of 44 (k = 11; n = 4) frankfurters, with 132 frankfurters per trial, 528 frankfurters total. Frankfurters were selected based on sell-by dates to ensure all franks had the same expiration date. Frankfurters were acquired no more than 24 h prior to the onset of each trial of the current study.

After the procurement of frankfurters but prior to the onset of the experiment (~24 h prior to onset), a random sample of frankfurters were chosen for *Listeria* screening. A frankfurter was aseptically removed from the commercial packaging and placed in a sterile sampling bag. Approximately, 100 mL of sterile neutralizing buffered peptone water (nBPW; USDA-FSIS, 2016) was directly poured over the frankfurter and was homogenized in a Stomacher^®^ 400 Circulator (Seward) at 200 rpm for 1 min, was spread plated (100 μL) onto Oxford agar (Himedia Company, West Chester, Pennsylvania, USA), and incubated inverted for 18 to 24 h at 37 °C. Additionally, no enrichments of the homogenates were prepared and the limit of detection of the current methodology was 100 CFU/g when there was no visible growth on the incubated Oxford agar. Therefore, only packages that were confirmed to contain less than 100 CFU/g of *Listeria* were utilized in the current study.

### Inocula preparation

As this project was a continued effort of the work done by Bodie et al. [11], there are numerous studies on *L*. *monocytogenes* EDG-e ecology, functionality, genetic and biochemical data available, as well as whole-genome sequencing data, the well-characterized *L*. *monocytogenes* EDG-e was used for this study [26]. Additionally, as this study was done as a screen to the potential of SBS, SL, and their combination as short-duration antimicrobial dips on inoculated franks in the U.S. and is a continued effort from Bodie et al. [11], only *L*. *monocytogenes* EDG-e was used in the current study. Initially, a frozen stock of *L*. *monocytogenes* EDG-e was streaked for isolation on Oxford agar and incubated aerobically at 37 °C for 24 hours. A single colony was transferred to 20 mL of fresh TSB and incubated in a shaking incubator (100 rpm) overnight at 37 °C. The overnight culture of *L*. *monocytogenes* EDG-e was centrifuged at 25,000 × g for 5 min and subsequently washed twice in equal volumes (20 mL) of sterile 1 × Phosphate Buffered Saline (PBS). After washing, the final pellet was resuspended in 20 mL of PBS. The cell density of the inoculum used throughout the study was between 8 and 9 Log_10_ CFU/mL.

### Preparation of short-antimicrobial dip solutions

The treatment concentrations utilized in the current study were directly developed from the determined MIC and MBC concentrations for SBS and SL. Working stock solutions were prepared for antimicrobial compounds SBS, [0.39%, and 0.78% (w/v)] (Jones Hamilton Company, USA), SL [0.78% and 1.56% (w/v)] (Alfa Aesar), and the combination [0.39% SBS + 0.39% SL, 0.78% SBS + 0.78% SL (w/v)] in tap water (TW). Solutions were also made using the hotdog water (HDW) remaining in the package after removing the franks. The HDW was combined to create a bulk homogenate and treatments using HDW [HDW+ 0.78% SBS, HDW+ 1.56% SL (w/v)] and their combination [HDW + 0.78 SBS+ 0.78 SL] were generated. After treatments were made (k = 11), one replicate of each treatment solution was analyzed for pH with a SympHony pH meter and probe (VWR International, Radnor, PA, USA). The pH of SBS (0.39 and 0.78%) and SL (0.78 and 1.56%) prior to constructing combinatory treatments are shown in Table 1.

**Table 1:** The pH of the different treatments used throughout the study.

Treatment	pH
SBS 0.39%	1.75
SBS 0.78%	1.59
SL 0.78%	7.37
SL 1.56%	6.83

One replicate of each treatment was analyzed for pH with a SympHony pH meter and probe. Treatment pH analyzation was done before combination of treatments.

### Inoculation of frankfurters

A total of 176 frankfurters were used per trial (n = 4, N = 176, 4 sampling times, and k = 11), with the experiment being conducted at 3 independent times (3 trials) for a total of 132 frankfurters per sampling day and 528 frankfurters being used in total during the onset of the current experiment. Recently obtained frankfurters (< 24 h from purchase) with an average weight 47 g per frankfurter were transferred to sterile collection bags (VWR, Radnor, Pennsylvania, USA). Per frankfurter, 1 mL of the freshly prepared inocula with a confirmed cell density between 8 and 9 Log_10_ CFU/mL was spot inoculated onto the surface of the frankfurters in a sterile collection bag. Inoculum levels were confirmed via serial dilution and spread plating on Oxford agar.

After the inoculation of the organic frankfurters, each group was stored at 4 °C for 60 min to allow for the attachment of the cells on the surface of the frankfurters. Using modified sample preparation and direct plating methods developed by the USDA-FSIS and FDA for *Listeria monocytogenes* in foods, the total inoculated *Listeria monocytogenes* EDG on both the surface and interior of the organic frankfurters, an inoculated, no treatment, control was weighed and subsequently stomached at 200 rpm for 1 min in 100 mL of nBPW [27, 28]. The homogenate was serially diluted in 1× PBS to 10^−6^, 100 μL of the diluted homogenate was spread plated on Oxford agar in duplicate, and incubated aerobically for 24 hours at 37 °C. The final attachment level was determined to be between 7 and 8 Log_10_ CFU/g.

### Treatment application

After the attachment period, the inoculated frankfurters were submerged in their respective antimicrobial treatments. Treatment applications of the eleven treatments (k = 11) were performed with one frank per treatment, with four replications (n = 4). Frankfurters were transferred to sterile wide-mouthed containers (500 mL capacity) with 20 mL of the respective antimicrobial solutions. Frankfurters were manually agitated for 10 sec, aseptically transferred to sterile collection bags, and allowed to rest for 2 minutes. Afterward, frankfurters were aseptically transferred to vacuum package bags, vacuum-sealed (VP 215, VacMaster, Greenville, SC, USA), and stored at 4 °C until bacterial enumeration could be conducted on days 0, 7, 14, and 21.

### *Listeria* enumeration

On days 0, 7, 14, and 21, treated frankfurters were removed from refrigeration (4 °C) and stomached for 1 min at 200 rpm in 100 mL of nBPW [29] so that injured, viable bacteria and biofilm-forming bacteria could be recovered without the continued action of the antimicrobials [30, 31]. The resulting homogenates were diluted 10^−1^ to 10^−6^, and were enumerated via direct plating methodology where 100 μL of the diluted homogenates were spread plated on Oxford agar in duplicate. Plates were aerobically incubated for 24 hours at 37 °C, with an enumeration range for this experiment being selected at 25 to 250 colonies per plate. Approximately 1 mL of the frankfurter homogenates collected on days 0, 7, 14, and 21 were aliquoted to 1.5 mL microcentrifuge tubes without transferring solid pieces of the frankfurters and stored at -20 °C until DNA extraction could be conducted.

### DNA extraction

Frankfurter homogenates collected on days 0, 7, 14, and 21 were thawed at room temperature, and the genomic DNA was extracted using the standard spin-column procedure for cultured cells using the QIAGEN DNeasy Blood & Tissue Kit (Qiagen, Valencia, CA, USA). Extracted samples were eluted in 50 μL of Buffer AE by directly hydrating the spin column and allowed to incubate at room temperature for 10 min. All eluted DNA samples were analyzed using a NanoDrop 1000 Spectrophotometer (Thermo Fisher Scientific, Waltham, MA, USA) to determine the isolated DNA concentration and purity ratios (260/280, 260/230). The extracted DNA was diluted to 10 ng/μL in Buffer AE and stored at -20°C until the library could be prepared.

### Library preparation

A sequencing library was constructed based on the V34, V4, and V45 regions of the 16S rDNA using custom primers designed by Kozich et al. [32]. Individual DNA samples were amplified with dual-indexed primers, including unique eight nucleotide barcode sequences, using a high-fidelity polymerase (Accuprime Pfx DNA polymerase, Thermo Fisher Scientific, Waltham, MA, USA) and verified using gel electrophoresis. Normalization was performed on PCR products in equimolar concentration (20 μL) using a SequalPrep^™^ Normalization kit (Life Technologies, Carlsbad, CA, USA). The pooled library contained a 5 μL aliquot of each normalized sample. Concentrations were determined using a KAPA library quantification kit for Illumina platforms (Kapa Biosystems, Woburn, MA, USA) and a Qubit fluorometer (Invitrogen, Carlsbad, CA, USA). At the same time, product size was assessed using an Agilent 2100 Bioanalyzer System (Agilent, Santa Clara, CA, USA). The library and PhiX Control v3 (Illumina, Carlsbad, CA, USA) were diluted to 20 nM in HT1 Buffer and denatured in 0.2 N fresh NaOH to generate a final concentration of 12 pM. The resulting library was combined with PhiX control v3 (20%, v/v) and loaded onto a MiSeq v2 (500 cycles) reagent cartridge (Illumina, Carlsbad, CA, USA). Subsequent sequences were uploaded to BaseSpace (Illumina, San Diego, California, USA), NCBI Sequence Read Archive (PRJNA732908), and GitHub (https://github.com/RickeLab-UW/CleanLabelAntimicrobialsOnFranks).

### Statistical and bioinformatic analyses

Each frankfurter was randomly assigned to treatment prior to the onset of the study. The CFU of *Listeria* was first Log_10_ transformed and reported on a CFU of *Listeria* per gram of frankfurter basis (CFU/g). Subsequently, the recovered *Listeria* per gram of frankfurter on d 7, 14, and 21 was subtracted by the initial recovered *Listeria* per gram of frankfurter on d 0 to represent the growth potential of *Listeria* per gram of frankfurter on d 7, 14, and 21. The data were analyzed in a mixed effect model with the random effect being trial and the fixed effects being time and treatment in RStudio. Version 1.4.1103 using the lme4, lmerTest, emmeans, and multcomp packages to determine the effect of treatment, day, or potential interaction between the two [33–37]. To further delineate differences between treatments, data were analyzed using One-way ANOVA in a mixed effects model, separated by day, with trial designated as the random effect. All means were separated using Tukey’s Protected HSD with a significant level of P ≤ 0.05.

The QIIME2 pipeline (version 2020.8) was utilized for sequencing data analyses [38]. Demultiplexed reads were downloaded from the Illumina BaseSpace website and were uploaded into Qiime2-2020.8 using Casava 1.8 paired-end demultiplexed format (via qiime tools import). Demultiplexed sequences were subjected to quality filtering and denoising in DADA2 via q2-dada2 [39]. The operational taxonomic units (OTU’s) were aligned with mafft, and a rooted phylogenetic tree was generated with fasttree2 (via q2-phylogeny) [40]. The OTUs were identified using SILVA (silva-138-99-nb-classifier.qza) [41–43] with the sk-learn Bayesian algorithm at a 95% confidence (via q2-feature-classifier) [44]. Subsequently, α- and β-diversity were generated via q2-diversity. Main effects and interactions were identified using ANOVA (via q2-longitudinal) [45] and ADONIS [46] for α- and β-diversity metrics. Pairwise comparisons of α-diversity metrics, Faith’s Phylogenetic Diversity, Shannon’s Diversity Index, Observed Features, and Pielou’s Evenness, were determined using Kruskal Wallis [47–49]. In addition, pairwise comparisons were determined for β-diversity metrics, Unweighted and Weighted Unifrac, Bray Curtis, and Jaccard, using ANOSIM [50–54]. Differentially abundant taxa were evaluated using ANCOM, the analysis of composition of microbiomes (via q2-composition) [55]. Final ANCOM tables and mean taxa were visualized in Microsoft Excel (Microsoft, Redmond, WA, USA). All metrics were analyzed without chloroplast and mitochondria being included using taxonomy-based filtering of tables and sequences (via q2-taxa). The main effects were significant at P ≤ 0.05 and pairwise differences when Q ≤ 0.05.

## Results

### pH of treatments

The solutions of SBS at 0.39 and 78% and SL at 0.78 and 1.56% were the only solutions measured for pH during the onset of the experiment (Table 1). The pH of the SBS solutions were 1.75 and 1.59 and the pH of the SL solutions were 7.37 and 6.83. The pH of the solutions was not statistically analyzed as there was insufficient replication of those being measured for pH.

### Mitigation of *Listeria*

The MIC in the current study was used to determine the range of antimicrobial concentrations that would inhibit the visible growth of *Listeria monocytogenes* EDG-e. Therefore, 0.39% SBS, 0.78% SL, and 0.39% SBS+SL were determined to be the minimum inhibitory concentrations and 0.78% SBS, 1.56% SL, and 0.781% SBS+SL were determined to be the minimal bacteriostatic concentrations that were ultimately used in the current experiment (S1 Fig). The pH was measured for SBS and SL prior to being combined. The pH levels of SBS treatments before combination were 1.75 for 0.39% SBS and 1.59 for 0.78% SBS and the pH levels of SL treatments before combination were 6.83 at 0.78% SL and 7.37 at 1.56% SL (Table 1).

Initially, a mixed effect model was utilized to determine the main effect of treatment and time and their subsequent interaction on the growth potential of *Listeria* (S2 Table). In the current study, there was an interaction between treatment and time, d 7, 14, and 21, on the growth potential of *Listeria* (P < 0.0001). The growth potential was lowest on d 7 compared to d 21 for most treatments; except those treated with SBS at 0.39% and HDW (HDW + SBS 0.78%, HDW + SL 1.56% and HDW + SBS + SL 0.78%). On d 7, the growth potential of *Listeria* from those treated with 1.56% SL was the lowest (-0.009 Log_10_ CFU/g), but by d 14 and 21 the growth potential of those frankfurters had increased to 0.0.628 and 0.569 Log_10_ CFU/g and did not have the lowest growth potential among treated frankfurters. In fact, on d 21, frankfurters treated with SBS 0.39% had the least numerical growth potential (0.361 Log_10_ CFU/g). The growth potential of those treated with SBS 0.39% on d 21 were not different than those treated with SBS 0.78% but were different than all other treatments on d 21. Overall, there was no change in the growth potential of *Listeria* of those treated with 0.39 and 0.78% of SBS from d 0 to d 21. Due to the complexity of the analysis, further analyses were performed to separate means by day to delineate day by day efficacy of treatments on the growth potential of *Listeria*.

**Fig 2:**
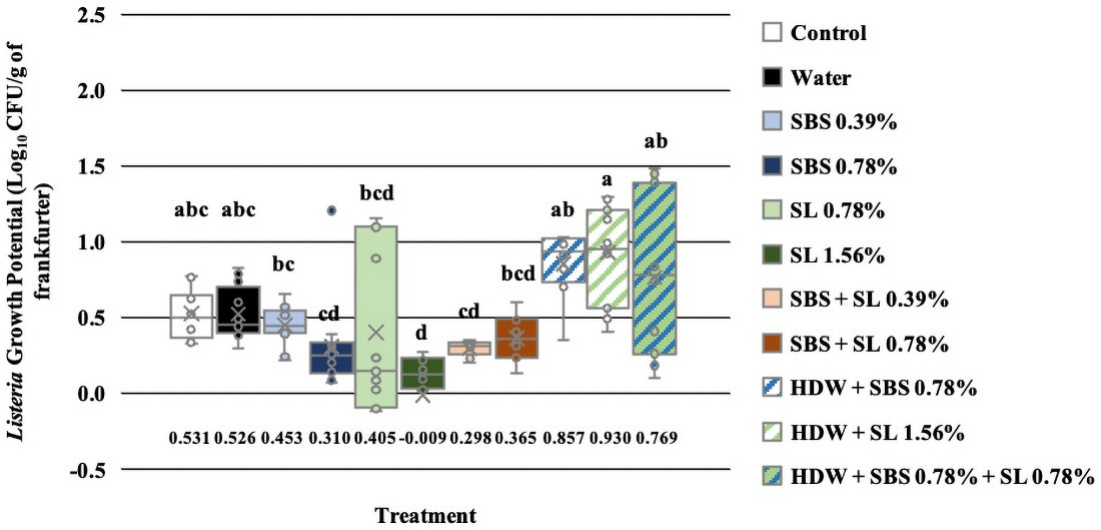
The growth potential of *Listeria* on frankfurters after antimicrobial treatment on d 7. Frankfurters were treated in 20 mL of the following treatments and their respective combinations for 10 sec, removed from treatments, allowed to rest for 2 min, and homogenized in 100 mL of nBPW for 1 min at 200 rpm. Controls were designated as a no treatment control and tap water. Treatments were comprised of sodium bisulfate (SBS) at 0.39 and 0.78%; sodium lactate (SL) at 0.78 and 1.56%; the combination of SBS and SL at 0.38 and 0.78%; and hotdog water (HDW) with the inclusion of either SBS (0.78%), SL (1.56%), or their combination (0.78%); Recovery was calculated by subtracting the Log_10_ CFU/g of each treatment on d 0 from d 7. Each letter annotates the significant difference between treatments. P < 0.0001, N = 132, and n = 44.

**Fig 3:**
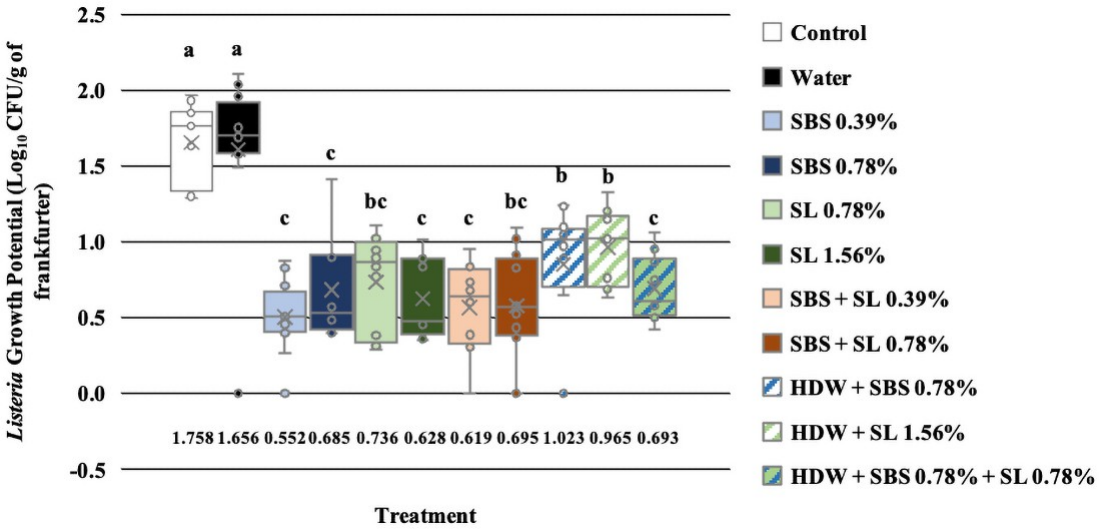
The growth potential of *Listeria* on frankfurters after antimicrobial treatment on d 14. Frankfurters were treated in 20 mL of the following treatments and their respective combinations for 10 sec, removed from treatments, allowed to rest for 2 min, and homogenized in 100 mL of nBPW for 1 min at 200 rpm. Controls were designated as a no treatment control and tap water. Treatments were comprised of sodium bisulfate (SBS) at 0.39 and 0.78%; sodium lactate (SL) at 0.78 and 1.56%; the combination of SBS and SL at 0.38 and 0.78%; and hotdog water (HDW) with the inclusion of either SBS (0.78%), SL (1.56%), or their combination (0.78%); Recovery was calculated by subtracting the Log_10_ CFU/g of each treatment on d 0 from d 14. Each letter annotates the significant difference between treatments. P < 0.0001, N = 132, and n = 44.

The effect of treatment was separated by day to delineate the effect of treatments on the growth potential of *Listeria* on d 7, 14, and 21, independently. On d 7, only those treated with SL at 1.56% (-0.009 Log_10_ CFU/g) had a significantly lower growth potential compared to the no treatment control and those treated with tap water (0.531 and 0.526 Log_10_, P < 0.05; Fig 2). The growth potential of *L*. *monocytogenes* EDG-e of frankfurters treated with SBS 0.78%, SL 0.79%, SBS + SL 0.39%, and SBS + SL 0.78% were not different than those treated with SL 1.56%. Unlike on d 7, the growth potential of *L*. *monocytogenes* EDG-e of treated frankfurters on d 14 was significantly less than that of the no treatment control and those treated with tap water (1.758 and 1.656 Log_10_ CFU/g, P < 0.05, Fig 3). Additionally, on d 14, the growth potential of those treated with SBS (0.39 and 0.78%), SL 1.56%, SBS + SL 0.39%, and HDW + SBS + SL 0.78% was less than that of the controls and those treated with HDW + SBS 0.78% and HDW + SL 1.56% but was not different to the growth potential of those treated with SL 0.78% and SBS + SL 0.78%. Numerically, the growth potential of *Listeria* of the frankfurters treated with SBS 0.39% (0.552 Log_10_ CFU/g) were the lowest compared to all other treatments even though they were not different to those treated with SBS 0.78%, SL 1.56%, SBS + SL 0.39%, and HDW + SBS + SL 0.78%.

**Fig 4:**
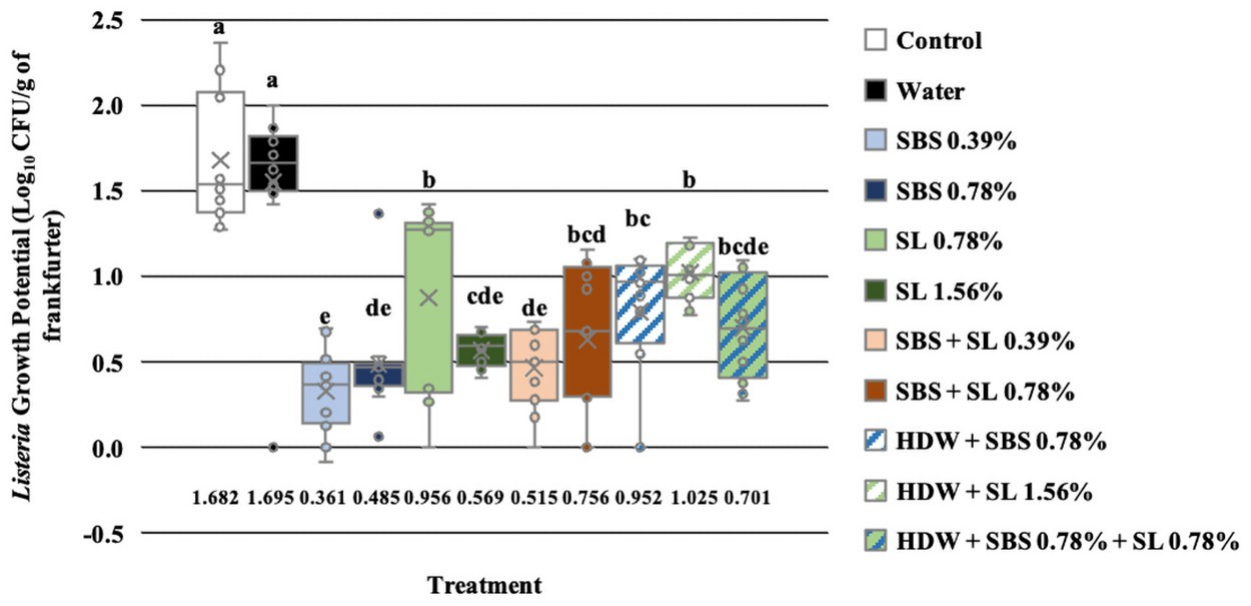
The growth potential of *Listeria* on frankfurters after antimicrobial treatment on d 21. Frankfurters were treated in 20 mL of the following treatments and their respective combinations for 10 sec, removed from treatments, allowed to rest for 2 min, and homogenized in 100 mL of nBPW for 1 min at 200 rpm. Controls were designated as a no treatment control and tap water. Treatments were comprised of sodium bisulfate (SBS) at 0.39 and 0.78%; sodium lactate (SL) at 0.78 and 1.56%; the combination of SBS and SL at 0.38 and 0.78%; and hotdog water (HDW) with the inclusion of either SBS (0.78%), SL (1.56%), or their combination (0.78%); Recovery was calculated by subtracting the Log_10_ CFU/g of each treatment on d 0 from d 21. Each letter annotates the significant difference between treatments. P < 0.0001, N = 132, and n = 44.

On d 21, all experimentally treated franks had less growth potential of *Listeria* compared to the no treatment control and TW treated franks (1.682 and 1.695 Log_10_ CFU/g; (P < 0.05; Fig 4). The lowest growth potential of *Listeria* was detected among those treated with the SBS at 0.39%; however, there was no difference between those treated with SBS 0.78%, SL 1.56%, SBS + SL 0.39%, and HDW + SBS + SL 0.78% and those treated with SBS at 0.39% (0.361 Log_10_ CFU/g). The growth potential of those treated with SBS 0.39% was different than the no treatment controls and those treated with water, SL 0.78%, SBS + SL 0.78%, HDW + SBS 0.78%, and HDW + SL 1.56. Additionally, the growth potential of those treated with SL 0.78% and HDW + SL 1.56% was not different than that of those treated with HDW + SBS + SL 0.78% and SBS + SL 0.78%.

### Impact of *Listeria* and treatment on the microbiota of frankfurters

**Fig 5:**
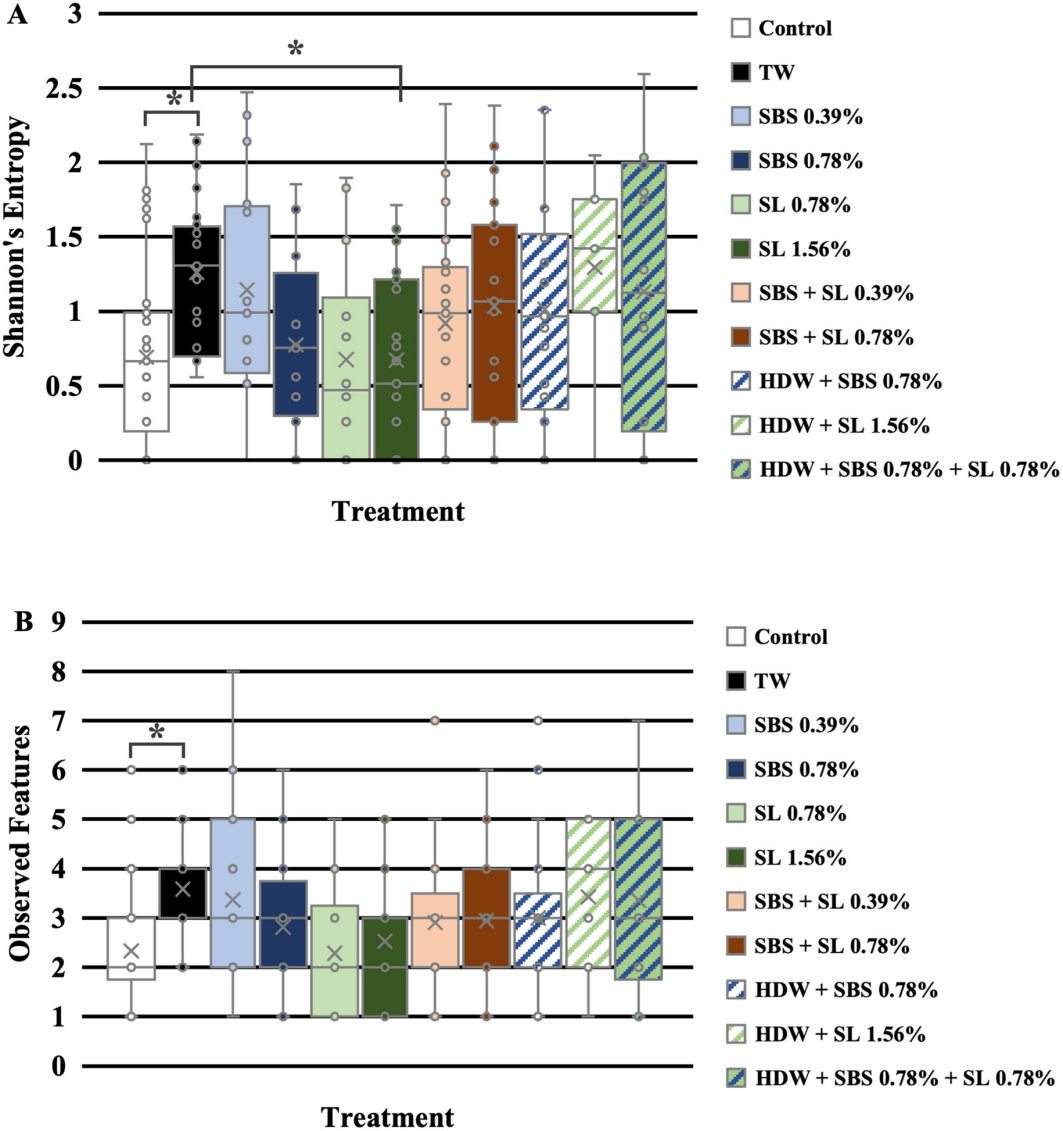
The main effect of treatment on Shannon’s Diversity (A) and Observed Features (B). Pairwise differences were determined by Kruskal-Wallis and pairwise significance is indicated with asterisks (P < 0.05; Q < 0.05).

Using ANOVA, the main effects and interactions were explored for α-diversity metrics on the indigenous frankfurter microbiota. The main effect of treatment was only significant for Shannon’s diversity (P = 0.029), and there was no main effect of time or interaction of treatment × time for any of the other α-diversity metrics (S3 Table; P > 0.05). There was a trending effect of treatment on the observed features of the frankfurter homogenate when using ANOVA in QIIME2 (P = 0.082). Using Kruskal-Wallis, the pairwise differences between the main effect of treatment were explored (S4 Table). The only significant differences observed between the Shannon’s entropy (Fig 5A; P = 0.023) of the treated frankfurters were among the homogenates of the frankfurters treated as the no treatment control and treated with TW (P = 0.001, Q = 0.044) and differences between those treated with TW and SL 1.56% (P = 0.002, Q = 0.044). Those treated with TW had a higher Shannon’s entropy than those treated as the control or with 1.56% SL. Although there was only a trending effect of treatment on the observed features of frankfurters (Fig 5B, P = 0.030), the main effect using Kruskal-Wallis was significant with significant pairwise differences occurring between the homogenates of the frankfurters treated with TW compared to those treated with the control (P < 0.001, Q = 0.012). Those treated with TW had greater observed features (OTU’s) than those treated as the control.

**Fig 6:**
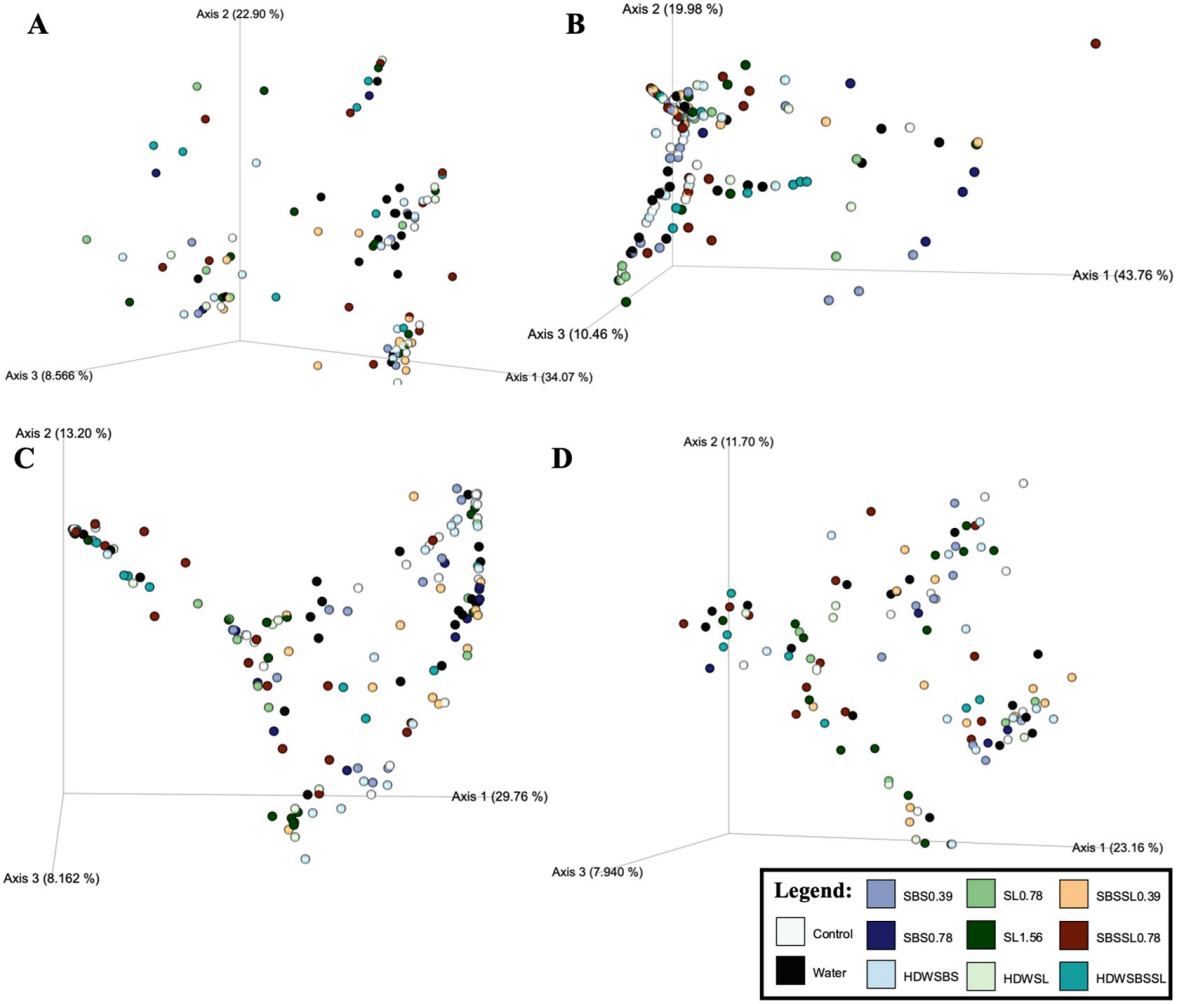
Beta diversity metrics: Unweighted Unifrac (A), Weighted Unifrac (B), Bray Curtis (C), and Jaccard (D). Metrics were performed using ANOSIM.

When using ADONIS, a non-parametric multivariate analysis of variance, there were no interactions between treatment and time on any of the β-diversity metrics (S5 Table, P > 0.05). However, there was a main effect of treatment for both Bray Curtis and Jaccard diversity metrics when using ADONIS (P = 0.001). To delineate the pairwise differences of the β-diversity metrics, ANOSIM, a non-parametric analysis of similarities based on ranked dissimilarities, was used (S6 Table, Fig 6). As such, the main effect of treatment and subsequent pairwise differences of Weighted Unifrac, Bray Curtis, and Jaccard (P = 0.001, 0.002, 0.001) were significantly different ANOSIM. Pairwise differences were observed between the homogenate of frankfurters treated with the control and that of all other antimicrobial treatments (P < 0.05; Q < 0.05). In addition, the Weighted Unifrac and Bray Curtis dissimilarity of the no treatment control frankfurters were different than that of those treated with both SL at 0.78% and HDW + SL at 1.56% (P < 0.05; Q < 0.05).

**Fig 7:**
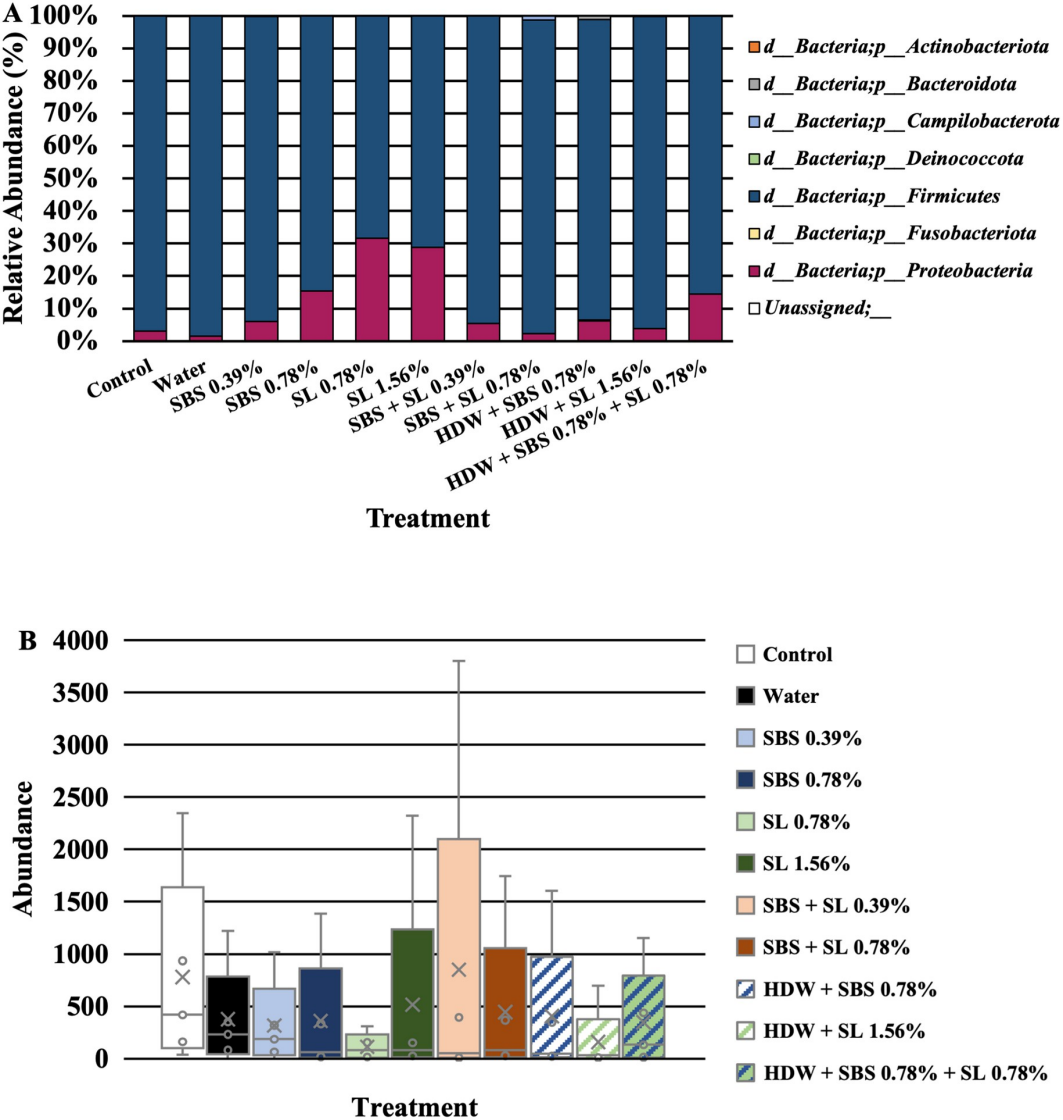
Mean taxa (A) and significantly different taxa as determined by ANCOM (B) at the phylum level when the taxa *Listeria* was included in the analyses. *Firmicutes* were the only significantly different taxa at the phylum level when using ANCOM (P < 0.05, W = 8).

**Fig 8:**
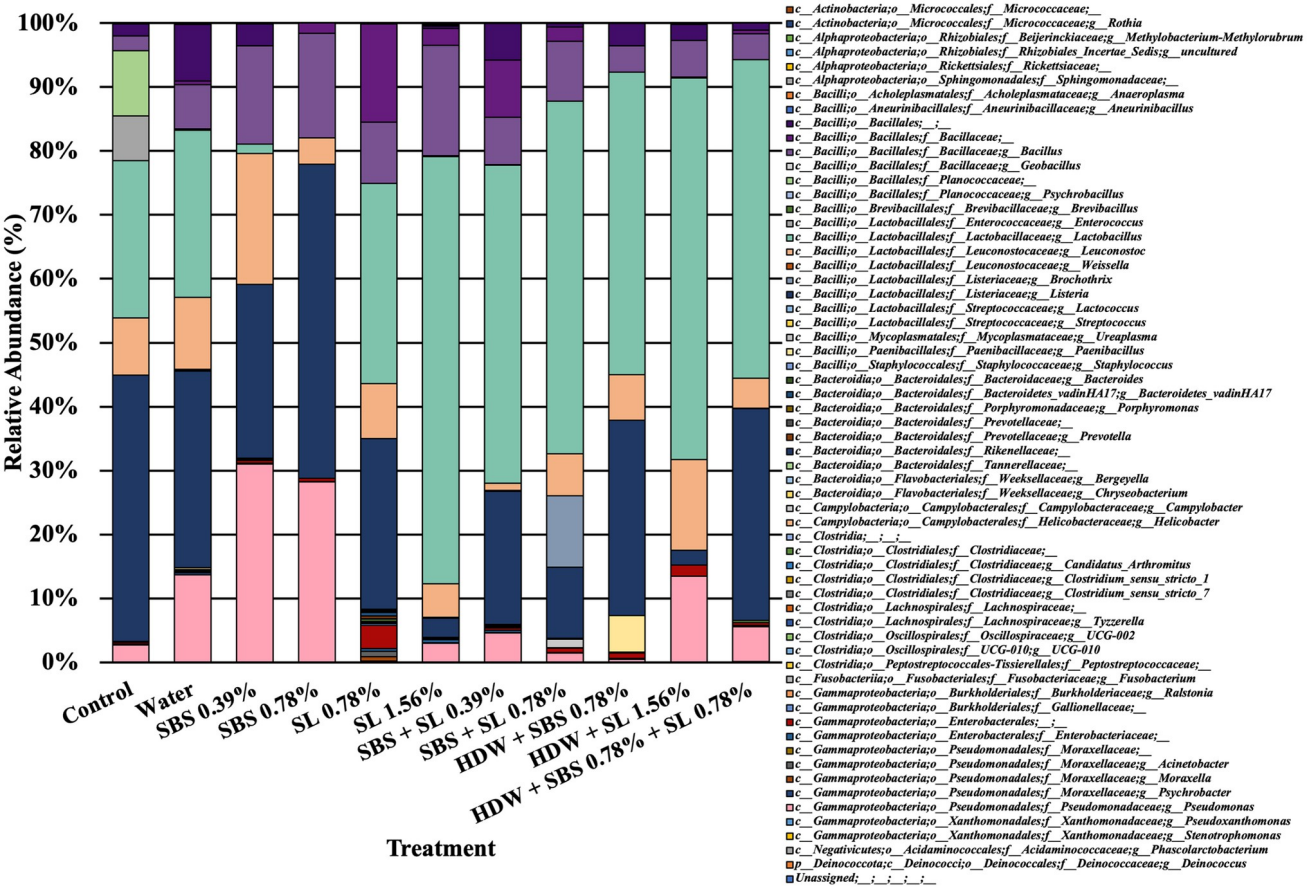
Mean taxa at the genus level.

**Fig 9:**
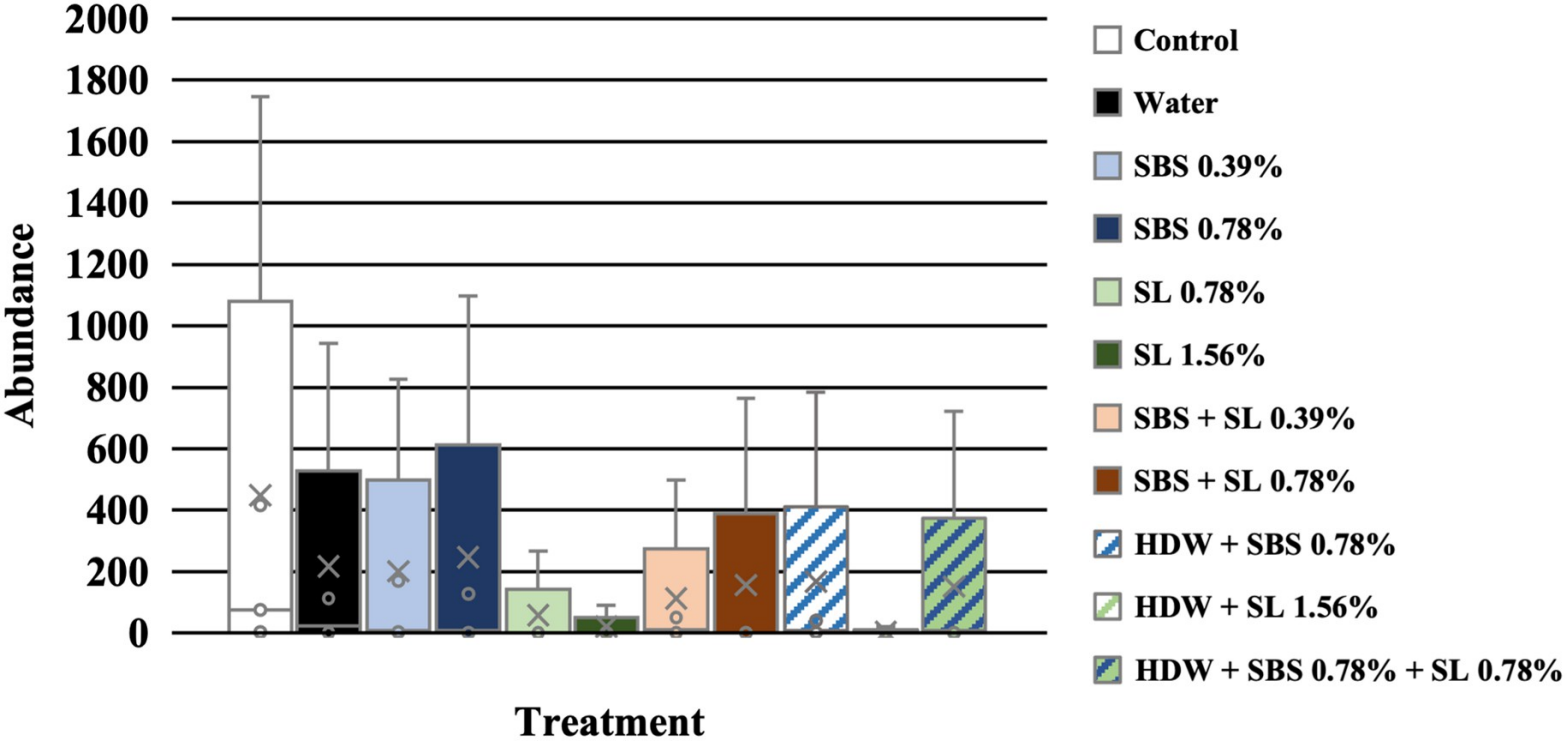
Significantly different taxa as determined by ANCOM at the genus level. *Listeria* were the only significantly different taxa at the genus level (P < 0.05, W = 52).

To look at the overall microbial composition of frankfurters inoculated with *Listeria monocytogenes*, mean taxa bar plots were generated. In addition, ANCOM was utilized to determine the significantly different abundant taxa among the microbiota at the phylum and genus levels (Figs 7–9). The three main taxa at the phyla level among the frankfurters were *Proteobacteria* and *Firmicutes*, although other phyla were detectable at much lower abundances (*Actinobacteriota*, *Bacteroidota*, *Campilobacterota*, *Deinococcota*, *Fusobacteriota*, and unassigned; Fig 7a). Firmicutes were the only significant differently abundant phyla identified through ANCOM (W = 7; Fig 7b). Of the mean genera, numerous taxa were present among the frankfurters treated in the current study such as: *Bacillales* (order), *Bacillaceae* (family), *Bacillus*, *Lactobacillus*, *Leuconostoc*, *Listeria*, *Pseudomonas* (Fig 8). Among the mean genera, those frankfurters treated with SBS at 0.39 or 0.78% levels had a numerically lower relative abundance of *Lactobacillus* and a higher relative abundance of *Pseudomonas* than any of the control or experimentally treated franks. However, the only significant genus was *Listeria* when using ANCOM (P < 0.05; W = 52, Fig 9). Although not significant, *Leuconostoc* had the second highest differently abundant value at the genus level (W = 2, P > 0.05).

## Discussion

As consumer education and awareness of chemical and synthetic additives increases, the demand for “healthier” alternative food products is also increasing [56]. This phenomenon has brought upon numerous changes in the food industry including the inclusion of “clean”, “natural”, and “organic” ingredients in RTE meats and products [57]. As such, there may be a need for multiple intervention steps during and post-production of these RTE meats. The purpose of this study was to compare the effect of potential alternative “organically acceptable” or “natural” antimicrobials, SBS and SL, as short duration dips on the mitigation of *Listeria monocytogenes* EDG-e and the impact of the microbiota of organic frankfurters during a 3-week shelf-life. Organic, no additives frankfurters were obtained from a local supermarket, confirmed *Listeria*-free, and artificially inoculated with *L*. *monocytogenes* EDG-e. There were 11 different short-antimicrobial dip treatments comprised of SBS and SL alone or in various combinations with tap water (TW) and hot dog water (HDW). Throughout the study (21-d), experimental short-antimicrobial dip treatments reduced *L*. *monocytogenes* EDG-e populations compared to the control groups, the no treatment control and TW. Although only *L*. *monocytogenes* EDG-e was utilized during this shelf-life study, this does provide potential insight into the use of these antimicrobials as short duration dips instead of being directly incorporated into the frankfurter batter as many antimicrobials have been added in the past.

### Acidity of treatments

The pH of both SBS and SL solutions were taken to determine how acidic these products were in solution. The pH of the SBS (0.39 and 0.78%) solutions was below 2; whereas the pH of SL (0.78 and 1.56%) solutions was close to neutral with a pH between 6.5 and 7.5. Although the pH of the solutions for the combinations was not taken, it can be noted that the pH of SBS was much lower than that of SL. Tthe pH of the SL solutions was 7.37 and 6.83 (0.78 and 1.56%, respectively). However, as there was minimal replication of the pH of the solutions and they were not statistically analyzed, nothing can be concluded.

### Rinsing effect of water on *Listeria*

The use of tap water in the current study was utilized as a control in addition to the no treatment control. There was no pre-treatment of the tap water in terms of sterilization as the tap water was directly obtained from the water supply at the Center for Food Safety at the University of Arkansas. The water supply at the University of Arkansas is derived from the Beaver Water District (Lowell, AR, USA) that is distributed to multiple cities and municipalities in Northwest Arkansas. According to the 2020 water quality report at the City of Fayetteville Arkansas which distributed the Beaver Water supply to the University of Arkansas, there is less than 1 ppm of chlorine, fluoride, nitrates, and less than 0.01 ppm of lead and copper [58]. Therefore, the water quality of the tap water should not have interfered with the efficacy of the antimicrobials. However, it cannot be ruled out there may have been a potential introduction of microorganisms as the tap water was not sterilized. The tap water was specifically not sterilized to mimic what may occur in an industry setting.

It has been well established in other food matrices such as poultry that there is a rinsing effect of water on the loosely attached microorganisms on the surface of the product [59–63]. In these studies, there has been a range of efficacy of rinsing products with water for the reduction of bacteria ranging from 0.4 to 1.1 Log_10_ CFU/g [60–63]. In the current study, there was a 1 Log_10_ CFU/g reduction of *Listeria* when frankfurters were treated with tap water compared to those designated as the no treatment control. This reduction was sustained over a 21-d shelf-life and is in agreement with previous research. Thus, there is a potential rinsing effect of tap water when used as a 10-s dip prior to packaging.

### Efficacy of SBS and SL in frankfurter exudative (HDW)

It is well established that the contamination of frankfurters and other RTE meats with *Listeria monocytogenes* can occur anywhere during processing due to the potential for reintroduction. However, current lethality steps such as cooking or smoking should eliminate microorganisms on or within the RTE product [64]. The re-contamination of these products typically occurs post-lethality during handling or post processing steps such as slicing or packaging [64]. As the re-contamination is occurring on the surface of the product, rather than within, *Listeria monocytogenes* can often be located on the surface and in the exudate of RTE products [65, 66].

Therefore, in the current study, SBS, SL, and their combination were added to a bulk homogenate of the exudate from the frankfurters (HDW) used throughout the study. The specific aim was to see if these chemical interventions could reduce the surface contamination of *L*. *monocytogenes* EDG-e on the frankfurters. The results indicated that on d 7 the growth potential of *L*. *monocytogenes* EDG-e on the frankfurters treated with HDW with either the addition of SBS or SL or their combination were not different from one another, nor the controls used in the current study, the no treatment control and tap water. On d 14 and 21, the *L*. *monocytogenes* EDG-e growth potential in frankfurters treated with HDW with either the addition of SBS, SL, or their combination were different than the controls and the growth potential of *L*. *monocytogenes* EDG-e of those treated with the combination of HDW, SBS, and SL (0.78%) and those treated with SBS (0.39%), the treatment with the lowest growth potential, were not different on these days. Neither the treatment of frankfurters with HDW + SBS (0.78%) or HDW + SL (0.78%) resulted in similar growth potentials to that of HDW + SBS (0.78%) + SL (0.78%) and SBS (0.39%). Therefore, the combined addition of SBS and SL in the frankfurter exudate may provide an additional intervention strategy to use in a multi-hurdle approach to reduce the *Listeria monocytogenes* levels among RTE meats comparative to that of using SBS (0.39%) alone. Further research incorporating these antimicrobials in the interior of the RTE packaging would need to be explored in order to fully assess the robustness of this application.

### Mitigation of *Listeria* with SBS and SL

In the current study, the authors examined the addition of SBS to short duration antimicrobial dips post-cooking prior to packaging to mitigate *L*. *monocytogenes* and other potential foodborne pathogens. As such on d 21, the treatment of frankfurters with 0.39% SBS as a short duration antimicrobial dip resulted in only a 0.361 Log_10_ CFU/g growth potential of *L*. *monocytogenes* from d 0 and almost a 4 Log_10_ CFU/g reduction of *Listeria* compared to the controls at that time. Interestingly, the use of 0.39% SBS (0.552 and 0.361 Log_10_ CFU/g) as a short duration antimicrobial dip on *L*. *monocytogenes* EDG-e inoculated frankfurters resulted in numerically greater mitigation of growth potential over a 21-d refrigeration period compared to the higher SBS solution, 0.78%, on d 14 and 21 (0.685 and 0.485 Log_10_ CFU/g). However, there were no statistical differences between the two concentrations throughout the study. Therefore, it would be more cost effective for industry personnel to use the lower SBS concentration of 0.39% without losing antimicrobial potency.

Although, there is limited information on the use of SBS as an antimicrobial ingredient or short duration antimicrobial dip on frankfurters, this does conform with previous research involving SBS used as a short duration dip. Kim et al. [67] demonstrated that the application of 1 and 3% SBS as short duration antimicrobial dips (2 min) reduced *L*. *monocytogenes* on artificially contaminated Granny Smith apples (10^6^ Log_10_ CFU/g) by 2 and 5 Log_10_ CFU/g over a 14 day period. More recently, Bodie et al. [11] demonstrated the mitigation of *L*. *monocytogenes* on organic beef frankfurters with the use of 0.75 and 1.5% SBS achieving over a 2 Log_10_ CFU/g reduction of inoculated frankfurters immediately after treatment. In addition, SBS has been reported to be an effective antimicrobial agent against *Salmonella* spp. in multiple environments such as poultry parts, rendered chicken fat, and poultry reuse water [15–18]. Lastly, as SBS is GRAS [12], a safer alternative choice antimicrobial posing little to no risk [13], is not directly incorporated into the product, and does not alter the final composition of the product, it may potentially serve as a multi-hurdle approach without interfering with labeling schematics.

Sodium lactate is a frequent food additive to meat and poultry products as a flavor enhancer as its use in organic RTE meat is approved by the USDA and National Organic Standards Board [23]. In the current study, the use of 0.78% and 1.56% SL as an antimicrobial rinse did not serve as a significant pH control agent as it only lowered the surrounding pH of the antimicrobial solution from 7.37 to 6.83. Although sodium lactate can alter the pH of the surrounding environment [68], its primary modes of action are disruption of the bacterial cell through intracellular acidification and reduced water activity of the substrate [69]. Sodium lactate can be added at 2 to 4% levels in the meat batter without altering the meat pH, which is essential as a reduction in meat pH could reduce water holding capacity and subsequent quality [70]. Although the pH or water holding capacity of frankfurters were not measured in the current study, the low concentration of SL and the short duration application of 10-s would not be expected to affect these parameters. In our study, when SL was used alone as a dip treatment at its highest concentration of 1.56%, the *L*. *monocytogenes* population had a growth potential of 0.756 Log_10_ CFU/g at 21 days post inoculation which was significantly less than that of those designated as the no treatment control or those treated with TW (Fig 4). Lungu and Johnson [71], using 6% sodium lactate as a coating on full fat turkey frankfurter pieces (1 g), reported a 2.8 Log_10_ CFU/g increase in *L*. *monocytogenes* (V7) after 21 days at 4 °C. Conversely, Glass et al. [72] found that sodium lactate as part of a bratwurst formulation contributed to the suppression of *Listeria* growth by only 0.16 CFU/ per package after 30 days. It is suspected that the addition of SL into frankfurters formulation can provide a bacteriostatic effect.

Currently, there is a need for alternative antimicrobials for food safety interventions in organic products in the RTE meat industry. In the meat industry, SL and sodium diacetate are commonly used antimicrobials to create a synergistic effect on meat products. Several studies have verified that the addition of SL and sodium diacetate combinations to the formulation of food products creates a synergistic inhibition of *L*. *monocytogenes* in cured meat and poultry products [72]. However, organic acid salts can be less effective as an antimicrobial ingredient in the batter of uncured products [72, 73]. Therefore, SBS, an inorganic acid, could be an alternative antimicrobial to be combined with other organically acceptable antimicrobials in a multiple hurdle approach for limiting food pathogens in uncured organic products. The authors of the current study investigated the potential synergistic effect between SBS and SL for controlling populations of *Listeria* on artificially inoculated frankfurters when used as antimicrobial rinses. However, in the current study, there was no additive effect of SBS and SL in combination at any concentration implying that there is not a synergistic benefit from the inclusion of both SBS and SL in a 10-s dip. Additionally, the increase in concentration from 0.39% to 0.78% of both SBS and SL when in combination did not improve the efficacy of this short duration dip in mitigating *L*. *monocytogenes* EDG-e growth in frankfurters. The increase in concentration from 0.39% to 0.78% of both SBS and SL when in combination resulted in numerically higher growth potential throughout the study, similar to what was seen with the increase in concentration from 0.39% to 0.78% of SBS used alone. Therefore, there was no additive effect of increasing the concentration of SBS past 0.39% on frankfurters inoculated with *L*. *monocytogenes* EDG-e. Potentially, when SBS is applied to frankfurters as a 10-s rinse, a plateau in antimicrobial efficacy is reached between 0.39% and 0.78% but further studies would need to confirm this in the contexts of SBS applied as short duration dips on frankfurters inoculated with *L*. *monocytogenes* EDG-e.

Ultimately, the treatment with the lowest recovery of *L*. *monocytogenes* population illustrates the antimicrobial with the greatest efficacy. Throughout the study, depending on the day, the antimicrobial effect was not consistent across treatments with frankfurters. However, those treated with SL 1.56%, SBS 0.39%, and SBS 0.39% had the least growth potential of *L*. *monocytogenes* on d 7, 14, and 21, respectively. As such, the various antimicrobial treatments demonstrated different peaks in performance at the different time points, leading to the variation observed when assessing the most effective treatment per day. These results indicate that SBS and SL exhibited an anti-listerial effect on organic frankfurters from day 0 to day 21, with SBS exhibiting a greater impact on the reduction of *Listeria* as shown by d 14 and 21 pairwise comparisons.

### Identified taxa of frankfurters across time

In addition, to looking at the mitigation of *L*. *monocytogenes* EDG-e in the current study, this study was designed to determine the shift of the microbiota of artificially contaminated frankfurters in response to the aforementioned antimicrobial treatments over a 21-d shelf life. To date, there is limited knowledge identifying the microbial communities established on organic RTE frankfurters other than the potential for spoilage microorganisms and *Listeria* spp. Determining other taxa present on organic RTE franks is still being investigated, and the current research to the best of our knowledge is one of the first attempts to identify these communities using 16S rDNA sequencing, especially those highly contaminated with *Listeria monocytogenes*. Previous research investigating the microbial diversity between various ground beef products produced in South Korea using 16S sequencing (V1-V3; Roche 454) determined that *Pseudomonas* was the most abundant species (45.1%) among ground beef samples; however, *Lactobacillus* (16.4%), *Acinetobacter* (8.87%), *Carnobacterium* (6.49%), and *Enhydrobacter* (4.56%) were also major constituents of the core microbiota of ground raw beef [74]. In another study, Bowers [75] investigated the microbial communities of ground beef and subsequent further processed beef products by sequencing the 16S rDNA via an Illumina MiSeq. Bowers [75] determined that a cured beef frankfurter consisted primarily of *Proteobacteria* (approximately 50%), *Firmicutes* (<25%, but >50%), *Bacteroidetes* (<10%), and *Actinobacteria* (<10%) at the phyla level. At the genus level, Bowers [75] identified *Pseudomonas* (~25%), *Psychrobacter* (<10%), *Acinetobacter* (<10%), and *Lactobacillus* (<5%) as the core microbiota, although there was a large proportion of the microbiota defined as “undefined” or “other.” Among the beef frankfurters in the current study, although they were not cured, *Firmicutes*, and *Proteobacteria* were among the most prominent phyla across time (data compiled from d 0, 7, 14, and 21 d), and *Listeria*, *Lactobacillus*, *Bacillus*, *Pseudomonas*, and *Leuconostoc* were among the most prominent genera of the microbiota.

To delineate the potential differences in taxa abundances among treated frankfurters, ANCOM was utilized at both the phylum and genus level across time. As such, there were differently abundant taxa at both the phyla and genus level with *Firmicutes* (phylum) and *Listeria* (genus) being significantly different than 7 and 52 other taxa, respectively (P < 0.05; W = 7, W = 52). Unsurprisingly, the microbiome (16S) data was heavily biased by the inoculation of frankfurters with *L*. *monocytogenes*, as demonstrated by the ANCOM results at both the phyla and genera level. These differences were expected as over 8 Log_10_ CFU/g of *L*. *monocytogenes* was attached to the frankfurters after inoculation on d 0. In addition, this data did indicate that all experimental treatments reduced the relative abundance of *Listeria* compared to the control which is in congruence with the microbiological plate data. As ANCOM does not produce pairwise comparisons but rather relatively different abundant taxa further inference is not plausible. However, numerically speaking, those treated with SL 1.56% and HDW + SL 1.56% appeared to have the least relative abundance of *Listeria* compared to all other treated frankfurters which did not not align with the microbiological data in the current study.

### Microbial shifts were in response to treatment and not time

In addition to delineating differences among the identified taxa, it was important to detect any potential shifts of the α- (within differences) and β- (between differences) diversity in the current study across time. Significant shifts over time were expected among these diversity metrics as overall microbial loads typically increase over the shelf life of a product. Weinroth et al. [76] demonstrated a decrease in Faith’s PD over time (0, 6, and 15 d of dark storage, and d 5 of retail display after 21 d of dark storage) of previously antimicrobial and antioxidant treated ground beef indicating a decrease in diversity in parallel with an increase in total microbial load over time. In addition, that same research demonstrated that there was no interaction between treatment and time rather that time and treatment were independently impacting the microbiota and time had no effect on the Weighted and Unweighted Unifrac [76]. In congruence, interactions between treatment and time and a significant effect of treatment were not detected on the current study. However, time did not impact the diversity of the microbiota. This lack of difference over time could be due to the sustained reduction of treated frankfurters compared to those treated as the control and with tap water.

Although time was not significant, treatment did impact the microbiota. As such, those treated with TW had higher observed features (OTUs) than those treated with the no treatment control and a higher Shannon’s entropy than those treated with the no treatment control and SL 1.56%. It could be hypothesized that those treated with water had a greater richness due to the microbiota of tap water alone. Previous reports in the U.S. have demonstrated that tap water has a distinct microbiome that can contribute to the microbiome of downstream applications [77]. However, as 16S rDNA sequencing cannot distinguish live versus dead bacteria at this time, in the context of the current experiment it was not possible to definitively determine if the microbiota of the tap water actively contributed to the microbiota of the franks. As the microbiota of the tap water used to create the antimicrobial dip solutions in the current study was not explored, it was not possible to determine the direct influence the tap water had on the frankfurter microbiota. However, as differences were observed between those treated with TW and the no treatment control, it could be an indication of this effect. Future studies should consider elucidating the microbiota of the tap water used to create antimicrobial dip solutions in addition to the microbiota of the frankfurters or matrices of interest.

The only differences observed among the β-diversity metrics of the treated frankfurters were between the control and those treated with either SL 1.56% or HDW + SL 1.56% (Weighted Unifrac and Bray Curtis). Thus, the unique taxa present on frankfurters treated with the control were not the same as those identified on frankfurters treated with SL 1.56% or HDW + SL 1.56% and those unique taxa were phylogenetically unique or disperse.

### Sodium lactate: Delicate balance between *Listeria* and lactic acid producing bacteria

Although β-diversity metrics display dissimilarities and differences of the microbiome and provide insight into the shifting microbiome, these metrics do not necessarily determine where these differences are occurring or what taxa are actively contributing to these shifts. Therefore, ANCOM is commonly utilized to further delineate differences. However, in the current study only *Listeria* was different among the genera as the data was heavily biased by the inoculation of *L*. *monocytogenes*. As such, it was unclear of where these differences existed between the control treated franks and those treated with SL 1.56% alone or treated with HDW + SL 1.56%. Inferring from the mean relative abundant taxa at the genus level, it can be seen that those treated with SL, especially those treated with 1.56% SL, yielded greater proportions of *Lactobacillus* than those treated as the control or treated with SBS alone at 0.39 and 0.78%. This high proportion of one taxa could have contributed to these differences in Weighted Unifrac and Bray Curtis but because this was not delineated in ANCOM it cannot be determined within the confines of this study. Previous work where SL was incorporated into beef sausage batter demonstrated the ability of 1.2 to 1.6% concentrations of SL to reduce lactic acid bacteria (LAB) over a 60-day shelf life [78]. However, as SL was not incorporated into the batter in the current study, but rather as a short duration antimicrobial dip it may not have been able to reduce the pH of the frank sufficiently to mitigate LAB commonly associated with spoilage since LAB are relatively tolerant to acidic environments and to short chain fatty acids [79].

### Sodium bisulfate: Potential counterbalance to *Listeria* but not *Pseudomonas*

Although not significant when using ANCOM, *Leuconostoc* had the second highest differently abundant value at the genus level (W = 2, P > 0.05). Interestingly, the mean relative abundance of *Leuconostoc* was higher among franks treated with SBS (0.39%) compared to the controls and treated franks (not statistically significantly). *Leuconostoc*, a lactic acid-producing bacterium (LAB), has been demonstrated to be antagonistic towards *L*. *monocytogenes* [80]. Using multiple model approaches, Baka et al. [80] determined that even with an initial low load (10^2^ CFU/g of *L*. *carnosum*), *L*. *carnosum* was capable of exhibiting a bacteriostatic/bactericidal effect on *L*. *monocytogenes* through the acidification of its surroundings and direct nutrient competition. Although, *Leuconostoc* was not quantitated during the study using microbiological or molecular methods, it is important to note that the treatments with a high relative abundance of *Leuconosto*c such as those treated with SBS had the lowest recovered levels of *L*. *monocytogenes*. As such, the treatment of franks with SBS 0.78% reduced *L*. *monocytogenes* by 3.72 Log_10_ CFU/g reduction of compared to those designated as the control on d 21. As a follow up to these observations, it would be of interest to examine the direct response of *Leuconostic* spp. to SBS in pure culture studies to determine if there is some inherent tolerance of SBS by this microorganism.

Among those treated with SBS at 0.39 and 0.78% there was also a higher numerical (not statistically significant) relative abundance of *Pseudomonas* among the mean relative abundant genera. Previously, Oh et al. [68] using sodium lactate (5 and 10%) and sodium diacetate (5 and 10%) as antimicrobial dips on *Pseudomonas aeruginosa* inoculated frankfurters and hams demonstrated the combination of 10% sodium lactate and 10% sodium diacetate or the acidified solution of 5 or 10% sodium lactate (pH 3) was able to reduce *Pseudomonas* on the RTE meats by 2 and 4 Log_10_ CFU/g, respectively. Although we did not quantitate *Pseudomonas* using microbial or molecular techniques and current 16S sequencing pipelines are qualitative, the results demonstrated a potential decrease of *Pseudomonas* among those treated with SL.

## Conclusions

According to USDA-FSIS, frankfurters are safe for consumption only for 2 weeks at 4 °C, refrigeration [80]. In the current study, through d 21, all treatments reduced the *L*. *monocytogenes* EDG-e population on frankfurters by at least 3 Log_10_ CFU/g, compared to no treatment control and 2 Log_10_ CFU/g compared to those treated with tap water alone. At the final time point, day 21, those treated with SBS 0.78%, had the lowest numerical recovery of L. monocytogenes EDG-e. On that same day, those treated with 0.39% of SBS had the least growth potential from d 0 (0.361 Log_10_ CFU/g). Although the growth potential of those treated with SBS 0.39% were no different than those treated with SBS 0.78%, SL 1.56%, and SBS + SL 0.39%, the growth potential of those treated with 0.39% SBS was at least 1 Log_10_ CFU/g less than that of the others. Additionally, this study demonstrated the potential for incorporating antimicrobial such as SBS and SL in the HDW or the exudate of frankfurters for control of *L*. *monocytogenes*. Throughout the experiment, no visible discoloration was observed, and no synergistic effects were demonstrated combining SBS and SL.

In addition, this study is one of the first attempts at determining the microbiota response of organic uncured beef frankfurters inoculated with *L*. *monocytogenes* EDG-e to short duration antimicrobial dip treatments applied post-lethality. Not only was the microbiota elucidated, but the treatment of the frankfurters with the experimental antimicrobial short duration dips was determined to be the key factor effecting the microbial shifts, with no effect of time. These results paired with the *L*. *monocytogenes* plate count data may provide evidence that these antimicrobials are effective at stabilizing the core microbiota irrespective of time. However, diversity metrics demonstrated there were differences between the TW treated franks and those treated as the control or with SL 1.56%. Although *Listeria* was the only significant different taxa using ANCOM, the diversity results are on par with the microbiota compositions of the different treated frankfurters. Ultimately, these results show the potential of these antimicrobials, SBS and SL, as short duration dips for the mitigation of *L*. *monocytogenes* and the potential for prolonged shelf life in refrigerated organic frankfurters. In addition, this study illustrates the potential utility of microbiome characterization as for assessing shelf-life microbial ecology of RTE products.

It is important to note that this study was conducted with one well studied strain, *L*. *monocytogenes* EDG-e, and did not follow the rigorous standards set by The French Agency for Food, Environmental and Occupational Health & Safety (ANSES), the European Union Reference Laboratory for *Listeria monocytogenes*, or the International Association for Food Protection [81–83]. By following these standards more closely by using a cocktail of *L*. *monocytogenes* strains, adapting these strains to the cold environment (4 °C) prior to the study, and measuring water activity and pH of the frankfurters may have allowed the current research to capture the varying responses of *L*. *monocytogenes* strains to stressful environments [84]. Although the current study did not take into account these standards, the authors believe that this work is insight into the potential for SBS, SL, and their combination for use as short antimicrobial dips on RTE products prior to packaging and the use of these antimicrobials in the exudate of frankfurters (HDW). Prior to the industry adapting these short-duration dips, more research will need to be conducting using these standards.

## Supporting information

S1 Table Nutritional facts of 100 g of “organic, all-natural” beef frankfurters (uncured, no-nitrate or nitrite-added, no preservatives, no by-products, fully cooked, vacuum packaged) 1 used in the current study. (DOCX)

S2 Table The interaction of time and day on the growth potential of recovered *L*. *monocytogenes* (P < 0.0001). (DOCX)

S3 Table Main effects and interactions using ANOVA of the α-diversity metrics of the rinsates of frankfurters inoculated with *Listeria monocytogenes* and subsequently dipped in various antimicrobial solutions. (DOCX)

S4 Table Pairwise differences using Kruskall-Wallis of the α-diversity metrics of the rinsates of frankfurters inoculated with *Listeria monocytogenes* and subsequently dipped in various antimicrobial solutions. (DOCX)

S5 Table Main effects and interactions using ADONIS of the β-diversity metrics of the rinsates of frankfurters inoculated with *Listeria monocytogenes* and subsequently dipped in various antimicrobial solutions. (DOCX)

S6 Table Pairwise differences using ANOSIM (999 permutations) of the β-diversity metrics of the rinsates of frankfurters inoculated with *Listeria monocytogenes* and subsequently dipped in various antimicrobial solutions. (DOCX)

S1 Fig Colorimetric results determining the minimum inhibitory (MIC) and minimum bactericidal concentrations (MBC) of sodium bisulfate, sodium lactate, and their combination. Results demonstrated that 0.195 and 0.391% of sodium bisulfate was inhibitory and bactericidal to *Listeria monocytogenes*, respectfully. Whereas 0.391 and 1.563% was inhibitory and bactericidal to *Listeria monocytogenes*, respectfully. The MIC and MBC of the combination of Sodium Bisulfate and Sodium Lactate was determined to be 0.391 and 0.781%.
(TIF)
