# Supplementary material for: Comparison of ready-to-eat “organic” antimicrobials, sodium bisulfate, and sodium lactate, on Listeria monocytogenes and the indigenous microbiome of organic uncured beef frankfurters stored under refrigeration for three weeks
Source: PLoS One. 2022 Jan 20;17(1):e0262167. doi: 10.1371/journal.pone.0262167 (PMC8775584; doi:10.1371/journal.pone.0262167)
Supplement: S1 Table — (DOCX) [file pone.0262167.s001.docx]

**S1. Table. Nutritional facts of 100 g of “organic, all-natural” beef frankfurters (uncured, no-nitrate or nitrite-added, no preservatives, no by-products, fully cooked, vacuum packaged)^1^ used in the current study.**

| Nutrients |  | Amount per 100 g^2^ | Daily Value (%)^3^ |
| --- | --- | --- | --- |
| Lipids, Carbohydrates, and Protein | Total Fat | 19 g | 26 |
|  | Saturated Fat | 9 g | 43 |
|  | Trans Fat | 0 g | 0 |
|  | Cholesterol | 53 mg | 17 |
|  | Sodium | 1064 mg | 47 |
|  | Total Carbohydrate | 0 g | 0 |
|  | Fiber | 0 g | 0 |
|  | Sugars | 0 g | 0 |
|  | Protein | 15 g | - |
| Vitamin & Minerals | Vitamin A | 0 mcg | 0 |
|  | Calcium | 9.6 mg | 0 |
|  | Iron | 1.5 mg | 9 |
|  | Potassium | 209 mg | 17 |

^1^Ingredients: Grass-fed beef, water, contains less than 2% of the following: sea salt, paprika, dehydrated onion, spices, nutmeg oil, and celery powder

^2^Contains 234 calories per 100 g of beef franks, each frank was ~47g

^3^Percent daily values are based on a 2,000 caloric diet
