# Supplementary material for: Comparison of ready-to-eat “organic” antimicrobials, sodium bisulfate, and sodium lactate, on Listeria monocytogenes and the indigenous microbiome of organic uncured beef frankfurters stored under refrigeration for three weeks
Source: PLoS One. 2022 Jan 20;17(1):e0262167. doi: 10.1371/journal.pone.0262167 (PMC8775584; doi:10.1371/journal.pone.0262167)
Supplement: S2 Table — (DOCX) [file pone.0262167.s002.docx]

**S2 Table**. **The interaction of time and day on the growth potential of *L. monocytogenes* (P < 0.0001).**

| Day | Treatment | Mean (Log_10_ CFU/g) | | + | SEM |
| --- | --- | --- | --- | --- | --- |
| 7 | Control | 0.531 | ^gh^ | + | 0.046 |
|  | Water | 0.526 | ^gh^ | + | 0.050 |
|  | SBS 0.39% | 0.453 | ^hi^ | + | 0.040 |
|  | SBS 0.78% | 0.310 | ^jk^ | + | 0.087 |
|  | SL 0.78% | 0.405 | ^ghijk^ | + | 0.162 |
|  | SL 1.56% | -0.009 | ^l^ | + | 0.154 |
|  | SBS + SL 0.39% | 0.298 | ^k^ | + | 0.015 |
|  | SBS + SL 0.78% | 0.365 | ^ij^ | + | 0.047 |
|  | HDW + SBS 0.78% | 0.857 | ^cd^ | + | 0.067 |
|  | HDW + SL 1.56% | 0.930 | ^bcd^ | + | 0.094 |
|  | HDW + SBS 0.78% + SL 0.78% | 0.769 | ^cde^ | + | 0.152 |
| 14 | Control | 1.628 | ^a^ | + | 0.077 |
|  | Water | 1.758 | ^a^ | + | 0.060 |
|  | SBS 0.39% | 0.552 | ^fg^ | + | 0.056 |
|  | SBS 0.78% | 0.685 | ^ef^ | + | 0.094 |
|  | SL 0.78% | 0.736 | ^cde^ | + | 0.091 |
|  | SL 1.56% | 0.628 | ^efg^ | + | 0.075 |
|  | SBS + SL 0.39% | 0.619 | ^efg^ | + | 0.072 |
|  | SBS + SL 0.78% | 0.695 | ^e^ | + | 0.080 |
|  | HDW + SBS 0.78% | 1.023 | ^b^ | + | 0.054 |
|  | HDW + SL 1.56% | 0.965 | ^bc^ | + | 0.072 |
|  | HDW + SBS 0.78% + SL 0.78% | 0.693 | ^e^ | + | 0.061 |
| 21 | Control | 1.619 | ^a^ | + | 0.102 |
|  | Water | 1.695 | ^a^ | + | 0.054 |
|  | SBS 0.39% | 0.361 | ^ijk^ | + | 0.070 |
|  | SBS 0.78% | 0.485 | ^ghi^ | + | 0.088 |
|  | SL 0.78% | 0.956 | ^bcd^ | + | 0.153 |
|  | SL 1.56% | 0.569 | ^fg^ | + | 0.029 |
|  | SBS + SL 0.39% | 0.515 | ^gh^ | + | 0.062 |
|  | SBS + SL 0.78% | 0.756 | ^cde^ | + | 0.109 |
|  | HDW + SBS 0.78% | 0.952 | ^bc^ | + | 0.054 |
|  | HDW + SL 1.56% | 1.025 | ^b^ | + | 0.050 |
|  | HDW + SBS 0.78% + SL 0.78% | 0.701 | ^e^ | + | 0.084 |

^1^Frankfurters were inoculated with *L. monocytogenes* followed by dip treatments in their respective antimicrobial solutions. Frankfurters were treated in the following treatments and their combinations, SBS = sodium bisulfate; SL = sodium lactate; HDW = frankfurters liquid content; SBS + SL= sodium bisulfate and sodium lactate; HDW+SBS= frankfurters liquid content and sodium bisulfate; HDW+SL= frankfurters liquid content; SL; HDW+SBS+SL= frankfurters liquid content, sodium bisulfate and sodium lactate.

^2^Means with different letters are significantly different (a-l).
