# Supplementary material for: Comparison of ready-to-eat “organic” antimicrobials, sodium bisulfate, and sodium lactate, on Listeria monocytogenes and the indigenous microbiome of organic uncured beef frankfurters stored under refrigeration for three weeks
Source: PLoS One. 2022 Jan 20;17(1):e0262167. doi: 10.1371/journal.pone.0262167 (PMC8775584; doi:10.1371/journal.pone.0262167)
Supplement: S3 Table — (DOCX) [file pone.0262167.s003.docx]

**S3** **Table. Main effects and interactions using ANOVA of the α-diversity metrics of the rinsates of frankfurters inoculated with Listeria monocytogenes and subsequently dipped in various “clean label” antimicrobial solutions.**

|  |  | Faith's PD | | | Shannon's Diversity | | | Observed Features | | | Pielou's Evenness | | |
| --- | --- | --- | --- | --- | --- | --- | --- | --- | --- | --- | --- | --- | --- |
|  | df | SS | F-value | P-value | SS | F-value | P-value | SS | F-value | P-value | SS | F-value | P-value |
| Treatment | 10 | 0.267 | 0.824 | 0.606 | 9.351 | 2.073 | **0.029** | 38.219 | 1.709 | 0.082 | 0.421 | 0.811 | 0.618 |
| Time | 1 | 0.001 | 0.044 | 0.834 | 0.001 | 0.002 | 0.966 | 0.376 | 0.168 | 0.682 | 0.029 | 0.552 | 0.459 |
| Treatment × Time | 10 | 0.434 | 1.338 | 0.214 | 3.308 | 0.733 | 0.693 | 16.518 | 0.739 | 0.687 | 0.478 | 0.922 | 0.515 |
| Residual | 179 | 5.806 | NaN | NaN | 80.766 | NaN | NaN | 400.323 | NaN | NaN | 7.575 | NaN | NaN |
