# Supplementary material for: Comparison of ready-to-eat “organic” antimicrobials, sodium bisulfate, and sodium lactate, on Listeria monocytogenes and the indigenous microbiome of organic uncured beef frankfurters stored under refrigeration for three weeks
Source: PLoS One. 2022 Jan 20;17(1):e0262167. doi: 10.1371/journal.pone.0262167 (PMC8775584; doi:10.1371/journal.pone.0262167)
Supplement: S4 Table — (DOCX) [file pone.0262167.s004.docx]

**S4 Table. Pairwise differences using Kruskall-Wallis of the α-diversity metrics of the rinsates of frankfurters inoculated with *Listeria monocytogenes* and subsequently dipped in various “clean label” antimicrobial solutions.**

|  |  | Faith's PD | | | Shannon's Diversity | | | Observed Features | | | Pielou's Evenness | | |
| --- | --- | --- | --- | --- | --- | --- | --- | --- | --- | --- | --- | --- | --- |
| Group 1 | Group 2 | H | P-value | Q-value | H | P-value | Q-value | H | P-value | Q-value | H | P-value | Q-value |
| Control (n=30) | HDW + SBS 0.78% (n=21) | 2.850 | 0.091 | 0.828 | 2.579 | 0.108 | 0.371 | 3.605 | 0.058 | 0.406 | 0.208 | 0.648 | 0.860 |
| Control (n=30) | HDW + SBS 0.78% + SL 0.78% (n=14) | 0.740 | 0.390 | 0.863 | 2.059 | 0.151 | 0.384 | 2.707 | 0.100 | 0.423 | 0.181 | 0.671 | 0.860 |
| Control (n=30) | HDW + SL 1.56% (n=7) | 2.872 | 0.090 | 0.828 | 4.279 | 0.039 | 0.272 | 3.367 | 0.067 | 0.406 | 1.750 | 0.186 | 0.764 |
| Control (n=30) | SBS 0.39% (n=16) | 3.220 | 0.073 | 0.828 | 4.635 | 0.031 | 0.272 | 3.619 | 0.057 | 0.406 | 1.226 | 0.268 | 0.860 |
| Control (n=30) | SBS 0.78% (n=16) | 0.098 | 0.754 | 0.967 | 0.138 | 0.710 | 0.831 | 0.349 | 0.555 | 0.780 | 0.054 | 0.817 | 0.941 |
| Control (n=30) | SBS + SL 0.39% (n=21) | 0.011 | 0.916 | 0.977 | 1.976 | 0.160 | 0.384 | 1.666 | 0.197 | 0.516 | 0.230 | 0.631 | 0.860 |
| Control (n=30) | SBS + SL 0.78% (n=19) | 2.411 | 0.120 | 0.828 | 2.592 | 0.107 | 0.371 | 2.756 | 0.097 | 0.423 | 0.493 | 0.483 | 0.860 |
| Control (n=30) | SL 0.78% (n=14) | 0.003 | 0.960 | 0.977 | 0.027 | 0.869 | 0.921 | 0.022 | 0.882 | 0.975 | 0.000 | 0.984 | 0.989 |
| Control (n=30) | SL 1.56% (n=19) | 0.782 | 0.377 | 0.863 | 0.004 | 0.950 | 0.956 | 0.388 | 0.533 | 0.780 | 0.801 | 0.371 | 0.860 |
| Control (n=30) | Water (n=24) | 7.688 | 0.006 | 0.306 | 10.301 | **0.001** | **0.044** | 13.625 | **0.000** | **0.012** | 0.284 | 0.594 | 0.860 |
| HDW + SBS 0.78% (n=21) | HDW + SBS 0.78% + SL 0.78% (n=14) | 0.041 | 0.840 | 0.977 | 0.178 | 0.673 | 0.831 | 0.131 | 0.717 | 0.839 | 0.067 | 0.796 | 0.941 |
| HDW + SBS 0.78% (n=21) | HDW + SL 1.56% (n=7) | 0.593 | 0.441 | 0.869 | 1.073 | 0.300 | 0.533 | 0.807 | 0.369 | 0.635 | 0.405 | 0.524 | 0.860 |
| HDW + SBS 0.78% (n=21) | SBS 0.39% (n=16) | 0.006 | 0.939 | 0.977 | 0.377 | 0.539 | 0.781 | 0.021 | 0.886 | 0.975 | 0.087 | 0.768 | 0.939 |
| HDW + SBS 0.78% (n=21) | SBS 0.78% (n=16) | 1.189 | 0.276 | 0.863 | 1.124 | 0.289 | 0.530 | 1.247 | 0.264 | 0.572 | 0.782 | 0.377 | 0.860 |
| HDW + SBS 0.78% (n=21) | SBS + SL 0.39% (n=21) | 1.401 | 0.237 | 0.863 | 0.163 | 0.687 | 0.831 | 0.152 | 0.697 | 0.839 | 0.258 | 0.612 | 0.860 |
| HDW + SBS 0.78% (n=21) | SBS + SL 0.78% (n=19) | 0.009 | 0.924 | 0.977 | 0.027 | 0.871 | 0.921 | 0.001 | 0.978 | 0.983 | 0.002 | 0.960 | 0.989 |
| HDW + SBS 0.78% (n=21) | SL 0.78% (n=14) | 1.433 | 0.231 | 0.863 | 1.967 | 0.161 | 0.384 | 2.467 | 0.116 | 0.426 | 0.385 | 0.535 | 0.860 |
| HDW + SBS 0.78% (n=21) | SL 1.56% (n=19) | 0.177 | 0.674 | 0.951 | 2.316 | 0.128 | 0.371 | 0.876 | 0.349 | 0.635 | 2.635 | 0.105 | 0.718 |
| HDW + SBS 0.78% (n=21) | Water (n=24) | 0.378 | 0.539 | 0.896 | 1.625 | 0.202 | 0.445 | 3.458 | 0.063 | 0.406 | 0.029 | 0.864 | 0.950 |
| HDW + SBS 0.78% + SL 0.78% (n=14) | HDW + SL 1.56% (n=7) | 0.313 | 0.576 | 0.896 | 0.114 | 0.736 | 0.831 | 0.006 | 0.939 | 0.983 | 0.364 | 0.546 | 0.860 |
| HDW + SBS 0.78% + SL 0.78% (n=14) | SBS 0.39% (n=16) | 0.028 | 0.868 | 0.977 | 0.011 | 0.917 | 0.952 | 0.000 | 0.983 | 0.983 | 0.006 | 0.938 | 0.989 |
| HDW + SBS 0.78% + SL 0.78% (n=14) | SBS 0.78% (n=16) | 0.731 | 0.392 | 0.863 | 1.320 | 0.251 | 0.483 | 1.245 | 0.265 | 0.572 | 2.367 | 0.124 | 0.757 |
| HDW + SBS 0.78% + SL 0.78% (n=14) | SBS + SL 0.39% (n=21) | 0.291 | 0.589 | 0.896 | 0.605 | 0.437 | 0.706 | 0.405 | 0.524 | 0.780 | 0.679 | 0.410 | 0.860 |
| HDW + SBS 0.78% + SL 0.78% (n=14) | SBS + SL 0.78% (n=19) | 0.040 | 0.841 | 0.977 | 0.121 | 0.728 | 0.831 | 0.233 | 0.629 | 0.805 | 0.002 | 0.961 | 0.989 |
| HDW + SBS 0.78% + SL 0.78% (n=14) | SL 0.78% (n=14) | 0.281 | 0.596 | 0.896 | 1.806 | 0.179 | 0.410 | 2.226 | 0.136 | 0.462 | 0.179 | 0.672 | 0.860 |
| HDW + SBS 0.78% + SL 0.78% (n=14) | SL 1.56% (n=19) | 0.000 | 0.985 | 0.985 | 2.320 | 0.128 | 0.371 | 1.338 | 0.247 | 0.572 | 3.473 | 0.062 | 0.718 |
| HDW + SBS 0.78% + SL 0.78% (n=14) | Water (n=24) | 0.155 | 0.694 | 0.954 | 0.111 | 0.739 | 0.831 | 0.328 | 0.567 | 0.780 | 0.324 | 0.569 | 0.860 |
| HDW + SL 1.56% (n=7) | SBS 0.39% (n=16) | 0.447 | 0.504 | 0.869 | 0.700 | 0.403 | 0.672 | 0.199 | 0.656 | 0.820 | 0.736 | 0.391 | 0.860 |
| HDW + SL 1.56% (n=7) | SBS 0.78% (n=16) | 2.589 | 0.108 | 0.828 | 3.403 | 0.065 | 0.351 | 1.992 | 0.158 | 0.462 | 2.792 | 0.095 | 0.718 |
| HDW + SL 1.56% (n=7) | SBS + SL 0.39% (n=21) | 1.833 | 0.176 | 0.863 | 2.465 | 0.116 | 0.371 | 1.115 | 0.291 | 0.572 | 1.963 | 0.161 | 0.764 |
| HDW + SL 1.56% (n=7) | SBS + SL 0.78% (n=19) | 0.443 | 0.506 | 0.869 | 0.524 | 0.469 | 0.715 | 0.681 | 0.409 | 0.682 | 0.600 | 0.439 | 0.860 |
| HDW + SL 1.56% (n=7) | SL 0.78% (n=14) | 1.813 | 0.178 | 0.863 | 2.987 | 0.084 | 0.371 | 2.734 | 0.098 | 0.423 | 0.956 | 0.328 | 0.860 |
| HDW + SL 1.56% (n=7) | SL 1.56% (n=19) | 0.910 | 0.340 | 0.863 | 4.146 | 0.042 | 0.272 | 2.011 | 0.156 | 0.462 | 4.606 | 0.032 | 0.718 |
| HDW + SL 1.56% (n=7) | Water (n=24) | 0.503 | 0.478 | 0.869 | 0.110 | 0.741 | 0.831 | 0.001 | 0.981 | 0.983 | 1.684 | 0.194 | 0.764 |
| SBS 0.39% (n=16) | SBS 0.78% (n=16) | 0.575 | 0.448 | 0.869 | 2.698 | 0.100 | 0.371 | 1.409 | 0.235 | 0.572 | 1.729 | 0.189 | 0.764 |
| SBS 0.39% (n=16) | SBS + SL 0.39% (n=21) | 1.701 | 0.192 | 0.863 | 0.273 | 0.602 | 0.807 | 0.272 | 0.602 | 0.794 | 0.600 | 0.438 | 0.860 |
| SBS 0.39% (n=16) | SBS + SL 0.78% (n=19) | 0.004 | 0.947 | 0.977 | 0.062 | 0.804 | 0.884 | 0.057 | 0.811 | 0.930 | 0.032 | 0.859 | 0.950 |
| SBS 0.39% (n=16) | SL 0.78% (n=14) | 1.085 | 0.298 | 0.863 | 4.090 | 0.043 | 0.272 | 2.739 | 0.098 | 0.423 | 1.056 | 0.304 | 0.860 |
| SBS 0.39% (n=16) | SL 1.56% (n=19) | 0.247 | 0.619 | 0.896 | 4.038 | 0.044 | 0.272 | 1.155 | 0.283 | 0.572 | 4.965 | 0.026 | 0.718 |
| SBS 0.39% (n=16) | Water (n=24) | 0.247 | 0.619 | 0.896 | 0.496 | 0.481 | 0.715 | 1.706 | 0.192 | 0.516 | 0.828 | 0.363 | 0.860 |
| SBS 0.78% (n=16) | SBS + SL 0.39% (n=21) | 0.019 | 0.890 | 0.977 | 0.569 | 0.451 | 0.708 | 0.402 | 0.526 | 0.780 | 0.395 | 0.530 | 0.860 |
| SBS 0.78% (n=16) | SBS + SL 0.78% (n=19) | 1.161 | 0.281 | 0.863 | 0.993 | 0.319 | 0.548 | 0.981 | 0.322 | 0.611 | 1.794 | 0.180 | 0.764 |
| SBS 0.78% (n=16) | SL 0.78% (n=14) | 0.016 | 0.900 | 0.977 | 0.194 | 0.660 | 0.831 | 0.349 | 0.555 | 0.780 | 0.117 | 0.732 | 0.915 |
| SBS 0.78% (n=16) | SL 1.56% (n=19) | 0.508 | 0.476 | 0.869 | 0.285 | 0.594 | 0.807 | 0.007 | 0.932 | 0.983 | 0.725 | 0.395 | 0.860 |
| SBS 0.78% (n=16) | Water (n=24) | 1.617 | 0.204 | 0.863 | 5.392 | 0.020 | 0.272 | 7.357 | 0.007 | 0.123 | 1.390 | 0.238 | 0.860 |
| SBS + SL 0.39% (n=21) | SBS + SL 0.78% (n=19) | 1.089 | 0.297 | 0.863 | 0.281 | 0.596 | 0.807 | 0.141 | 0.708 | 0.839 | 0.220 | 0.639 | 0.860 |
| SBS + SL 0.39% (n=21) | SL 0.78% (n=14) | 0.082 | 0.774 | 0.967 | 1.369 | 0.242 | 0.483 | 1.199 | 0.274 | 0.572 | 0.051 | 0.821 | 0.941 |
| SBS + SL 0.39% (n=21) | SL 1.56% (n=19) | 0.478 | 0.490 | 0.869 | 1.405 | 0.236 | 0.483 | 0.265 | 0.606 | 0.794 | 2.655 | 0.103 | 0.718 |
| SBS + SL 0.39% (n=21) | Water (n=24) | 3.443 | 0.064 | 0.828 | 3.279 | 0.070 | 0.351 | 4.289 | 0.038 | 0.406 | 0.000 | 0.989 | 0.989 |
| SBS + SL 0.78% (n=19) | SL 0.78% (n=14) | 0.867 | 0.352 | 0.863 | 2.039 | 0.153 | 0.384 | 1.977 | 0.160 | 0.462 | 0.337 | 0.562 | 0.860 |
| SBS + SL 0.78% (n=19) | SL 1.56% (n=19) | 0.103 | 0.748 | 0.967 | 2.328 | 0.127 | 0.371 | 0.806 | 0.369 | 0.635 | 3.201 | 0.074 | 0.718 |
| SBS + SL 0.78% (n=19) | Water (n=24) | 0.086 | 0.769 | 0.967 | 1.296 | 0.255 | 0.483 | 2.551 | 0.110 | 0.426 | 0.262 | 0.609 | 0.860 |
| SL 0.78% (n=14) | SL 1.56% (n=19) | 1.008 | 0.315 | 0.863 | 0.003 | 0.956 | 0.956 | 0.341 | 0.559 | 0.780 | 1.315 | 0.252 | 0.860 |
| SL 0.78% (n=14) | Water (n=24) | 2.880 | 0.090 | 0.828 | 7.274 | 0.007 | 0.128 | 8.678 | 0.003 | 0.089 | 0.301 | 0.583 | 0.860 |
| SL 1.56% (n=19) | Water (n=24) | 1.031 | 0.310 | 0.863 | 9.979 | **0.002** | **0.044** | 6.634 | 0.010 | 0.138 | 4.118 | 0.042 | 0.718 |
