# Supplementary material for: Comparison of ready-to-eat “organic” antimicrobials, sodium bisulfate, and sodium lactate, on Listeria monocytogenes and the indigenous microbiome of organic uncured beef frankfurters stored under refrigeration for three weeks
Source: PLoS One. 2022 Jan 20;17(1):e0262167. doi: 10.1371/journal.pone.0262167 (PMC8775584; doi:10.1371/journal.pone.0262167)
Supplement: S5 Table — (DOCX) [file pone.0262167.s005.docx]

**S5 Table**. **Main effects and interactions using ADONIS of the β-diversity metrics of the rinsates of frankfurters inoculated with *Listeria monocytogenes* and subsequently dipped in various “clean label” antimicrobial solutions.**

|  |  | Unweighted Unifrac | | | | | Weighted Unifrac | | | | | Bray Curtis | | | | | Jaccard | | | | |
| --- | --- | --- | --- | --- | --- | --- | --- | --- | --- | --- | --- | --- | --- | --- | --- | --- | --- | --- | --- | --- | --- |
|  | Df | SS | MS | F-value | R2 | P-value | SS | MS | F-value | R2 | P-value | SS | MS | F-value | R2 | P-value | SS | MS | F-value | R2 | P-value |
| Treatment | 10 | 0.429 | 0.043 | 1.140 | 0.056 | 0.257 | 0.152 | 0.015 | 1.385 | 0.067 | 0.092 | 6.971 | 0.697 | 2.003 | 0.096 | **0.001** | 6.379 | 0.638 | 1.757 | 0.086 | **0.001** |
| Time | 1 | 0.026 | 0.026 | 0.690 | 0.003 | 0.600 | 0.009 | 0.009 | 0.828 | 0.004 | 0.464 | 0.378 | 0.378 | 1.085 | 0.005 | 0.355 | 0.332 | 0.332 | 0.913 | 0.004 | 0.492 |
| Treatment × Time | 10 | 0.439 | 0.044 | 1.166 | 0.058 | 0.240 | 0.137 | 0.014 | 1.249 | 0.061 | 0.194 | 3.159 | 0.316 | 0.908 | 0.043 | 0.670 | 2.810 | 0.281 | 0.774 | 0.038 | 0.953 |
| Residuals | 179 | 6.740 | 0.038 | NaN | 0.883 | NaN | 1.961 | 0.011 | NaN | 0.868 | NaN | 62.288 | 0.348 | NaN | 0.856 | NaN | 64.992 | 0.363 | NaN | 0.872 | NaN |
| Total | 200 | 7.634 | NaN | NaN | 1.000 | NaN | 2.259 | NaN | NaN | 1.000 | NaN | 72.796 | NaN | NaN | 1.000 | NaN | 74.512 | NaN | NaN | 1.000 | NaN |
