# Supplementary material for: Comparison of ready-to-eat “organic” antimicrobials, sodium bisulfate, and sodium lactate, on Listeria monocytogenes and the indigenous microbiome of organic uncured beef frankfurters stored under refrigeration for three weeks
Source: PLoS One. 2022 Jan 20;17(1):e0262167. doi: 10.1371/journal.pone.0262167 (PMC8775584; doi:10.1371/journal.pone.0262167)
Supplement: S6 Table — (DOCX) [file pone.0262167.s006.docx]

**S6 Table. Pairwise differences using ANOSIM (999 permutations) of the β-diversity metrics of the rinsates of frankfurters inoculated with *Listeria monocytogenes* and subsequently dipped in various “clean label” antimicrobial solutions.**

|  |  |  | Unweighted Unifrac | | | Weighted Unifrac | | | Bray Curtis | | | Jaccard | | |
| --- | --- | --- | --- | --- | --- | --- | --- | --- | --- | --- | --- | --- | --- | --- |
| Group 1 | Group 2 | n | R | P-value | Q-value | R | P-value | Q-value | R | P-value | Q-value | R | P-value | Q-value |
| Control (n=30) | HDW + SBS 0.78% (n=21) | 51 | 0.066 | 0.062 | 0.341 | 0.053 | 0.100 | 0.226 | 0.011 | 0.277 | 0.354 | 0.005 | 0.361 | 0.414 |
| Control (n=30) | HDW + SBS 0.78% + SL 0.78% (n=14) | 44 | 0.187 | 0.013 | 0.174 | 0.185 | 0.022 | 0.121 | 0.098 | 0.073 | 0.149 | 0.110 | 0.053 | 0.153 |
| Control (n=30) | HDW + SL 1.56% (n=7) | 37 | 0.361 | 0.009 | 0.165 | 0.591 | **0.001** | **0.028** | 0.329 | **0.004** | **0.055** | 0.283 | 0.011 | 0.106 |
| Control (n=30) | SBS 0.39% (n=16) | 46 | 0.080 | 0.094 | 0.431 | 0.159 | 0.014 | 0.110 | 0.040 | 0.223 | 0.309 | 0.029 | 0.245 | 0.306 |
| Control (n=30) | SBS 0.78% (n=16) | 46 | 0.120 | 0.032 | 0.227 | 0.156 | 0.031 | 0.140 | 0.061 | 0.149 | 0.228 | 0.078 | 0.100 | 0.185 |
| Control (n=30) | SBS + SL 0.39% (n=21) | 51 | 0.107 | 0.017 | 0.174 | 0.037 | 0.152 | 0.255 | 0.021 | 0.233 | 0.309 | 0.051 | 0.098 | 0.185 |
| Control (n=30) | SBS + SL 0.78% (n=19) | 49 | 0.151 | 0.006 | 0.165 | 0.199 | 0.002 | 0.037 | 0.188 | 0.001 | 0.028 | 0.147 | 0.004 | 0.106 |
| Control (n=30) | SL 0.78% (n=14) | 44 | 0.146 | 0.033 | 0.227 | 0.203 | 0.005 | 0.064 | 0.166 | 0.007 | 0.076 | 0.177 | 0.014 | 0.106 |
| Control (n=30) | SL 1.56% (n=19) | 49 | 0.163 | 0.006 | 0.165 | 0.209 | **0.001** | **0.028** | 0.194 | **0.001** | **0.028** | 0.166 | 0.003 | 0.106 |
| Control (n=30) | Water (n=24) | 54 | 0.076 | 0.019 | 0.174 | 0.077 | 0.022 | 0.121 | 0.026 | 0.146 | 0.228 | 0.037 | 0.080 | 0.176 |
| HDW + SBS 0.78% (n=21) | HDW + SBS 0.78% + SL 0.78% (n=14) | 35 | 0.006 | 0.385 | 0.588 | 0.021 | 0.256 | 0.343 | 0.042 | 0.145 | 0.228 | 0.038 | 0.147 | 0.225 |
| HDW + SBS 0.78% (n=21) | HDW + SL 1.56% (n=7) | 28 | 0.072 | 0.207 | 0.519 | 0.384 | 0.007 | 0.064 | 0.291 | 0.009 | 0.076 | 0.278 | 0.009 | 0.106 |
| HDW + SBS 0.78% (n=21) | SBS 0.39% (n=16) | 37 | -0.028 | 0.728 | 0.793 | 0.050 | 0.123 | 0.242 | 0.034 | 0.179 | 0.259 | 0.022 | 0.229 | 0.293 |
| HDW + SBS 0.78% (n=21) | SBS 0.78% (n=16) | 37 | -0.027 | 0.736 | 0.793 | 0.076 | 0.058 | 0.191 | 0.063 | 0.077 | 0.151 | 0.048 | 0.116 | 0.203 |
| HDW + SBS 0.78% (n=21) | SBS + SL 0.39% (n=21) | 42 | 0.025 | 0.141 | 0.456 | 0.004 | 0.332 | 0.406 | -0.017 | 0.640 | 0.704 | 0.018 | 0.220 | 0.293 |
| HDW + SBS 0.78% (n=21) | SBS + SL 0.78% (n=19) | 40 | -0.018 | 0.645 | 0.771 | 0.003 | 0.386 | 0.452 | 0.072 | 0.036 | 0.094 | 0.063 | 0.040 | 0.138 |
| HDW + SBS 0.78% (n=21) | SL 0.78% (n=14) | 35 | -0.010 | 0.489 | 0.640 | 0.039 | 0.219 | 0.313 | 0.119 | 0.028 | 0.090 | 0.119 | 0.028 | 0.110 |
| HDW + SBS 0.78% (n=21) | SL 1.56% (n=19) | 40 | 0.014 | 0.242 | 0.532 | 0.037 | 0.099 | 0.226 | 0.094 | 0.022 | 0.081 | 0.091 | 0.022 | 0.106 |
| HDW + SBS 0.78% (n=21) | Water (n=24) | 45 | 0.009 | 0.304 | 0.578 | -0.011 | 0.612 | 0.635 | -0.003 | 0.421 | 0.493 | 0.016 | 0.213 | 0.293 |
| HDW + SBS 0.78% + SL 0.78% (n=14) | HDW + SL 1.56% (n=7) | 21 | -0.016 | 0.517 | 0.661 | 0.206 | 0.046 | 0.169 | 0.164 | 0.031 | 0.090 | 0.129 | 0.076 | 0.174 |
| HDW + SBS 0.78% + SL 0.78% (n=14) | SBS 0.39% (n=16) | 30 | 0.009 | 0.308 | 0.578 | 0.021 | 0.222 | 0.313 | 0.104 | 0.040 | 0.096 | 0.094 | 0.045 | 0.146 |
| HDW + SBS 0.78% + SL 0.78% (n=14) | SBS 0.78% (n=16) | 30 | 0.010 | 0.292 | 0.578 | 0.054 | 0.081 | 0.212 | 0.130 | 0.020 | 0.081 | 0.113 | 0.019 | 0.106 |
| HDW + SBS 0.78% + SL 0.78% (n=14) | SBS + SL 0.39% (n=21) | 35 | 0.042 | 0.217 | 0.519 | 0.098 | 0.059 | 0.191 | 0.052 | 0.090 | 0.161 | 0.048 | 0.122 | 0.203 |
| HDW + SBS 0.78% + SL 0.78% (n=14) | SBS + SL 0.78% (n=19) | 33 | -0.034 | 0.750 | 0.793 | -0.023 | 0.636 | 0.648 | -0.044 | 0.875 | 0.908 | -0.046 | 0.856 | 0.888 |
| HDW + SBS 0.78% + SL 0.78% (n=14) | SL 0.78% (n=14) | 28 | -0.019 | 0.632 | 0.771 | -0.007 | 0.514 | 0.565 | 0.008 | 0.314 | 0.384 | 0.011 | 0.285 | 0.341 |
| HDW + SBS 0.78% + SL 0.78% (n=14) | SL 1.56% (n=19) | 33 | -0.032 | 0.729 | 0.793 | -0.018 | 0.600 | 0.635 | -0.057 | 0.935 | 0.952 | -0.068 | 0.983 | 0.994 |
| HDW + SBS 0.78% + SL 0.78% (n=14) | Water (n=24) | 38 | -0.002 | 0.457 | 0.628 | -0.010 | 0.489 | 0.549 | 0.019 | 0.236 | 0.309 | 0.025 | 0.227 | 0.293 |
| HDW + SL 1.56% (n=7) | SBS 0.39% (n=16) | 23 | 0.111 | 0.133 | 0.456 | 0.096 | 0.154 | 0.255 | 0.258 | 0.011 | 0.076 | 0.245 | 0.020 | 0.106 |
| HDW + SL 1.56% (n=7) | SBS 0.78% (n=16) | 23 | 0.087 | 0.172 | 0.498 | 0.095 | 0.162 | 0.255 | 0.262 | 0.016 | 0.081 | 0.222 | 0.025 | 0.106 |
| HDW + SL 1.56% (n=7) | SBS + SL 0.39% (n=21) | 28 | 0.183 | 0.086 | 0.430 | 0.457 | 0.006 | 0.064 | 0.290 | 0.019 | 0.081 | 0.274 | 0.019 | 0.106 |
| HDW + SL 1.56% (n=7) | SBS + SL 0.78% (n=19) | 26 | 0.015 | 0.399 | 0.591 | 0.197 | 0.073 | 0.212 | 0.084 | 0.172 | 0.256 | 0.111 | 0.120 | 0.203 |
| HDW + SL 1.56% (n=7) | SL 0.78% (n=14) | 21 | 0.060 | 0.214 | 0.519 | 0.074 | 0.210 | 0.312 | -0.006 | 0.454 | 0.520 | 0.010 | 0.352 | 0.412 |
| HDW + SL 1.56% (n=7) | SL 1.56% (n=19) | 26 | -0.008 | 0.471 | 0.632 | 0.085 | 0.200 | 0.306 | -0.046 | 0.704 | 0.759 | -0.033 | 0.629 | 0.678 |
| HDW + SL 1.56% (n=7) | Water (n=24) | 31 | 0.113 | 0.111 | 0.436 | 0.225 | 0.040 | 0.157 | 0.183 | 0.022 | 0.081 | 0.122 | 0.088 | 0.181 |
| SBS 0.39% (n=16) | SBS 0.78% (n=16) | 32 | 0.007 | 0.317 | 0.578 | 0.037 | 0.147 | 0.255 | 0.007 | 0.313 | 0.384 | 0.014 | 0.263 | 0.321 |
| SBS 0.39% (n=16) | SBS + SL 0.39% (n=21) | 37 | 0.079 | 0.051 | 0.312 | 0.105 | 0.033 | 0.140 | 0.055 | 0.088 | 0.161 | 0.091 | 0.040 | 0.138 |
| SBS 0.39% (n=16) | SBS + SL 0.78% (n=19) | 35 | -0.001 | 0.408 | 0.591 | 0.038 | 0.116 | 0.236 | 0.100 | 0.022 | 0.081 | 0.078 | 0.049 | 0.150 |
| SBS 0.39% (n=16) | SL 0.78% (n=14) | 30 | 0.046 | 0.119 | 0.436 | 0.008 | 0.314 | 0.393 | 0.099 | 0.027 | 0.090 | 0.120 | 0.025 | 0.106 |
| SBS 0.39% (n=16) | SL 1.56% (n=19) | 35 | 0.014 | 0.272 | 0.575 | 0.010 | 0.308 | 0.393 | 0.051 | 0.106 | 0.182 | 0.032 | 0.191 | 0.269 |
| SBS 0.39% (n=16) | Water (n=24) | 40 | -0.025 | 0.640 | 0.771 | 0.010 | 0.340 | 0.407 | 0.022 | 0.234 | 0.309 | 0.037 | 0.152 | 0.226 |
| SBS 0.78% (n=16) | SBS + SL 0.39% (n=21) | 37 | 0.008 | 0.335 | 0.578 | 0.049 | 0.107 | 0.226 | 0.042 | 0.137 | 0.228 | 0.040 | 0.144 | 0.225 |
| SBS 0.78% (n=16) | SBS + SL 0.78% (n=19) | 35 | 0.002 | 0.378 | 0.588 | 0.093 | 0.030 | 0.140 | 0.144 | 0.004 | 0.055 | 0.096 | 0.021 | 0.106 |
| SBS 0.78% (n=16) | SL 0.78% (n=14) | 30 | 0.004 | 0.350 | 0.578 | 0.055 | 0.096 | 0.226 | 0.127 | 0.010 | 0.076 | 0.129 | 0.012 | 0.106 |
| SBS 0.78% (n=16) | SL 1.56% (n=19) | 35 | -0.026 | 0.775 | 0.804 | 0.022 | 0.162 | 0.255 | 0.048 | 0.091 | 0.161 | 0.028 | 0.180 | 0.261 |
| SBS 0.78% (n=16) | Water (n=24) | 40 | -0.032 | 0.741 | 0.793 | 0.081 | 0.080 | 0.212 | 0.082 | 0.055 | 0.121 | 0.085 | 0.069 | 0.173 |
| SBS + SL 0.39% (n=21) | SBS + SL 0.78% (n=19) | 40 | 0.022 | 0.182 | 0.501 | 0.073 | 0.019 | 0.121 | 0.092 | 0.017 | 0.081 | 0.063 | 0.061 | 0.168 |
| SBS + SL 0.39% (n=21) | SL 0.78% (n=14) | 35 | -0.006 | 0.439 | 0.619 | 0.078 | 0.104 | 0.226 | 0.095 | 0.064 | 0.135 | 0.074 | 0.089 | 0.181 |
| SBS + SL 0.39% (n=21) | SL 1.56% (n=19) | 40 | 0.003 | 0.357 | 0.578 | 0.042 | 0.079 | 0.212 | 0.057 | 0.051 | 0.117 | 0.052 | 0.069 | 0.173 |
| SBS + SL 0.39% (n=21) | Water (n=24) | 45 | 0.031 | 0.119 | 0.436 | 0.025 | 0.134 | 0.253 | 0.003 | 0.355 | 0.424 | 0.000 | 0.383 | 0.430 |
| SBS + SL 0.78% (n=19) | SL 0.78% (n=14) | 33 | -0.043 | 0.850 | 0.866 | -0.008 | 0.484 | 0.549 | -0.005 | 0.473 | 0.531 | -0.019 | 0.626 | 0.678 |
| SBS + SL 0.78% (n=19) | SL 1.56% (n=19) | 38 | -0.016 | 0.686 | 0.793 | -0.010 | 0.600 | 0.635 | -0.029 | 0.811 | 0.858 | -0.023 | 0.758 | 0.802 |
| SBS + SL 0.78% (n=19) | Water (n=24) | 43 | 0.011 | 0.351 | 0.578 | 0.016 | 0.250 | 0.343 | 0.077 | 0.033 | 0.091 | 0.034 | 0.143 | 0.225 |
| SL 0.78% (n=14) | SL 1.56% (n=19) | 33 | -0.047 | 0.875 | 0.875 | -0.038 | 0.799 | 0.799 | -0.072 | 0.992 | 0.992 | -0.070 | 0.994 | 0.994 |
| SL 0.78% (n=14) | Water (n=24) | 38 | 0.036 | 0.227 | 0.520 | 0.023 | 0.307 | 0.393 | 0.106 | 0.038 | 0.095 | 0.075 | 0.076 | 0.174 |
| SL 1.56% (n=19) | Water (n=24) | 43 | 0.033 | 0.154 | 0.471 | 0.034 | 0.138 | 0.253 | 0.080 | 0.031 | 0.090 | 0.045 | 0.101 | 0.185 |
