# Supplementary figures and images for: Comparison of ready-to-eat “organic” antimicrobials, sodium bisulfate, and sodium lactate, on Listeria monocytogenes and the indigenous microbiome of organic uncured beef frankfurters stored under refrigeration for three weeks
Source: PLoS One. 2022 Jan 20;17(1):e0262167. doi: 10.1371/journal.pone.0262167 (PMC8775584; doi:10.1371/journal.pone.0262167)

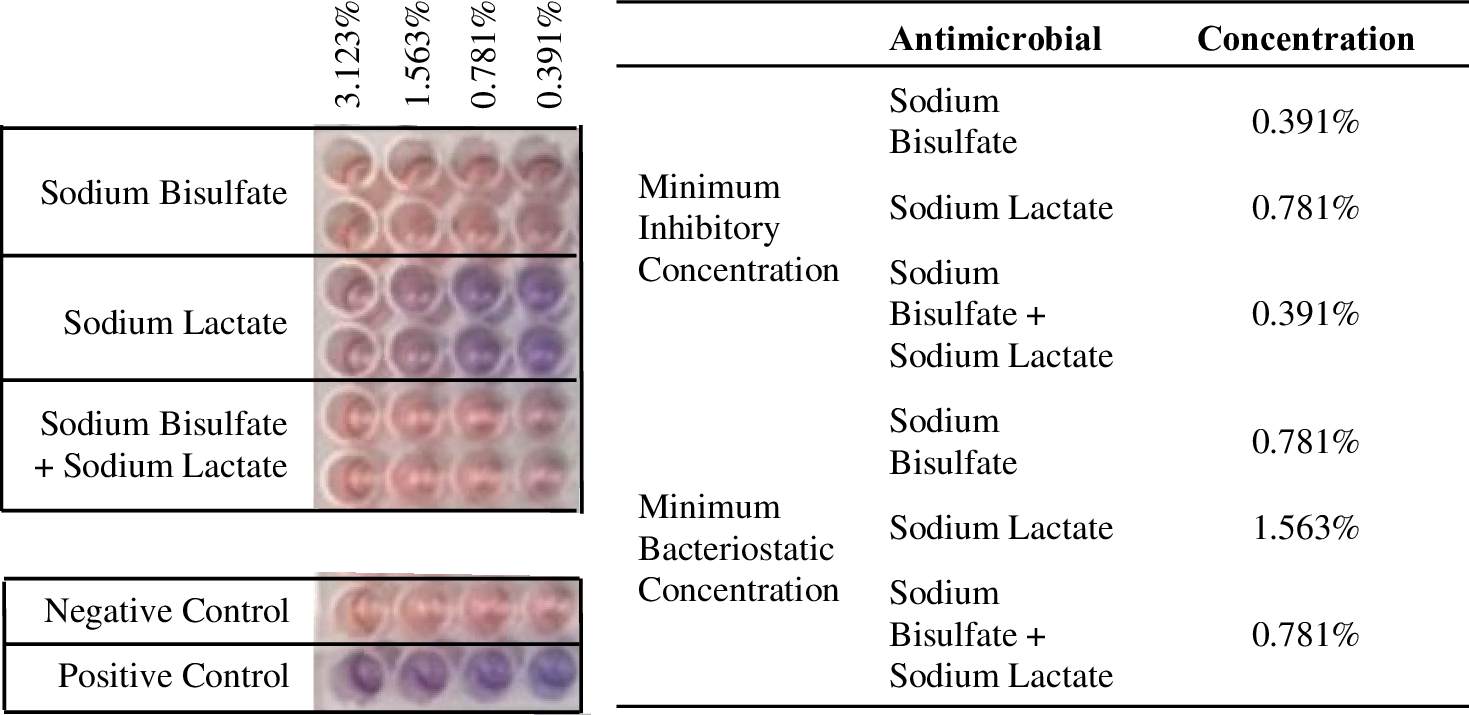

Supplement: S1 Fig — Results demonstrated that 0.195 and 0.391% of sodium bisulfate was inhibitory and bactericidal to Listeria monocytogenes, respectfully. Whereas 0.391 and 1.563% was inhibitory and bactericidal to Listeria monocytogenes, respectfully. The MIC and MBC of the combination of Sodium Bisulfate and Sodium Lactate was determined to be 0.391 and 0.781%. (TIF) [file pone.0262167.s007.tif]
